# Force-induced tail-autotomy mitochondrial fission and biogenesis of matrix-excluded mitochondrial-derived vesicles for quality control

**DOI:** 10.1073/pnas.2217019121

**Published:** 2024-03-28

**Authors:** Xiaoying Liu, Linyu Xu, Yutong Song, Zhihao Zhao, Xinyu Li, Cheuk-Yiu Wong, Rong Chen, Jianxiong Feng, Yitao Gou, Yajing Qi, Hei-Man Chow, Shuhuai Yao, Yi Wang, Song Gao, Xingguo Liu, Liting Duan

**Affiliations:** ^a^Department of Biomedical Engineering, The Chinese University of Hong Kong, Hong Kong SAR 999077, China; ^b^Department of Chemical and Biological Engineering, The Hong Kong University of Science and Technology, Hong Kong SAR 999077, China; ^c^State Key Laboratory of Oncology in South China, Collaborative Innovation Center for Cancer Medicine, Sun Yat-sen University Cancer Center, Guangzhou 510060, China; ^d^Department of Physics, The Chinese University of Hong Kong, Hong Kong SAR 999077, China; ^e^School of Life Sciences, Faculty of Science, The Chinese University of Hong Kong, Hong Kong SAR 999077, China; ^f^Gerald Choa Neuroscience Institute, The Chinese University of Hong Kong, Hong Kong SAR 999077, China; ^g^Nexus of Rare Neurodegenerative Diseases, The Chinese University of Hong Kong, Hong Kong SAR 999077, China; ^h^Department of Mechanical and Aerospace Engineering, The Hong Kong University of Science and Technology, Hong Kong SAR 999077, China; ^i^Chinese Academy of Sciences Key Laboratory of Regenerative Biology, Guangdong Provincial Key Laboratory of Stem Cell and Regenerative Medicine, China-New Zealand Joint Laboratory on Biomedicine and Health, Chinese University of Hong Kong-Guangzhou Institutes of Biomedicine and Health (CUHK-GIBH) Joint Research Laboratory on Stem Cells and Regenerative Medicine, Guangzhou Institutes of Biomedicine and Health, Chinese Academy of Sciences, Guangzhou Medical University, Guangzhou 510000, China; ^j^Centre for Regenerative Medicine and Health, Hong Kong Institute of Science & Innovation, Chinese Academy of Sciences, Hong Kong SAR 999077, China

**Keywords:** optogenetics, mitochondrial fission, photoactivatable proteins, tensile force, mitochondrial quality control

## Abstract

Mitochondria constantly fuse and divide for mitochondrial inheritance and functions. Here, we identified a distinct type of naturally occurring fission, tail-autotomy fission, wherein a tail-like thin tubule protrudes from the mitochondrial body and disconnects, resembling autotomy. Next, utilizing an optogenetic mitochondria-specific mechanostimulator, we revealed that mechanical tensile force drives tail-autotomy fission. This force-induced fission involves DRP1/MFF and endoplasmic reticulum tubule wrapping. It redistributes mitochondrial DNA, producing mitochondrial fragments with or without mitochondrial DNA for different fates. Moreover, tensile force can decouple outer and inner mitochondrial membranes, pulling out matrix-excluded tubule segments. Subsequent tail-autotomy fission separates the matrix-excluded tubule segments into matrix-excluded mitochondrial-derived vesicles (MDVs) which recruit Parkin and LC3B, indicating the unique role of tail-autotomy fission in segregating only outer membrane components for mitophagy. Sustained force promotes fission and MDV biogenesis more effectively than transient one. Our results uncover a mechanistically and functionally distinct type of fission and unveil the role of tensile forces in modulating fission and MDV biogenesis for quality control, underscoring the heterogeneity of fission and mechanoregulation of mitochondrial dynamics.

## Significance

Mitochondria are double-membranous organelles critical for cell survival and death. They constantly fuse and divide to maintain mitochondrial functions. Defective mitochondrial fission is implicated in cancers, cardiovascular diseases, and neurodegeneration. However, the heterogeneous nature, varied mechanisms, and roles of fission remain incompletely understood. Here, we identified a mechanistically and functionally distinct type of fission that initiates with the protrusion of a tail-like tubule followed by its disconnection, resembling tail autotomy. The tail-autotomy fission is driven by tensile force and can shed exclusively the outer membrane into matrix-excluded vesicles that can recruit mitophagic proteins for further degradation. Longer force application causes more fission and generation of matrix-excluded vesicles. These findings provide insights into the heterogeneity and mechanoregulation of mitochondrial fission.

Mitochondria continuously undergo fusion and fission, which shape the mitochondrial network, control intermitochondrial interactions, and maintain mitochondrial functions (1). Mitochondrial fission, by which a mitochondrion divides into two smaller units, had long been assumed to occur randomly along the length axis. However, a recent seminal report revealed two types of morphologically and mechanistically distinct fission which leverage different fission machineries and split the mitochondria at different submitochondrial locations (2). Midzone fission divides mitochondria near the center for mitochondrial biogenesis, while peripheral fission occurs near one end for mitophagy, highlighting the importance of investigating the determinants and outcomes of fission according to different fission categories. Nevertheless, whether there is any other type of fission contributing to the heterogeneity of mitochondrial fission remains elusive.

Fission is orchestrated by many executors and regulators. The pool of players and the mechanistic models for fission have been continuously expanding. Emerging evidence indicates the mechanoregulation of mitochondrial fission. Stiff extracellular matrix (3), mechanical stretching or increased pressure exerted on cells in vitro (4–6), and mechanical ventilation or mechanical needle stabbing of cells in vivo (7, 8) have been implicated in elevated mitochondrial fission and fragmentation. Furthermore, compressive force has been shown to directly trigger mitochondrial fission by compressing mitochondria with a motile bacterium colliding against mitochondria, the tip of atomic force microscopy, or uneven surfaces (9). On the other hand, tensile forces have been found to actively modulate mitochondrial morphology and fusion by driving the protrusion of thin, highly dynamic tubules out of the mitochondrial body (10, 11). Such tensile forces can be provided naturally inside the cells by other moving organelles or directly by molecular motors (10, 11). However, whether and how tensile forces play a role in mitochondrial fission is unknown.

Here by examining spontaneous mitochondrial dynamics, we identified a distinct type of mitochondrial fission, denoted as tail-autotomy fission, which is featured by two consecutive naturally occurring steps. First, a tail-like thin tubule extends out from the bulkier body. Next, the tubule gets disconnected, resembling the autotomy of the tail. Such tail-autotomy fission constitutes around 6 to 14% of all naturally occurring fission events in different types of cells. Next, utilizing an optogenetic mitochondria-specific mechanostimulator we recently developed (12), we unveiled the role of tensile forces in driving tail-autotomy fission. Upon light-gated exertion of pulling forces, a long thin tubule rapidly protrudes from the mitochondrial body and then undergoes autotomy-like division, demonstrating the causal linkage between tensile forces and tail-autotomy fission. MFF/DRP1 and endoplasmic reticulum (ER) tubule wrapping are involved in tail-autotomy fission. Tail-autotomy fission generates tubule fragments with or without mitochondrial DNA (mtDNA) for different fates. Moreover, we found that during force-induced tubulation, the outer mitochondrial membrane (OMM) and the inner mitochondrial membrane (IMM) can be decoupled, generating tubule segments composed of only OMM and forming matrix-excluded mitochondrial-derived vesicles (MDVs) by subsequent tail-autotomy division. Those matrix-excluded MDVs recruit Parkin and later the microtubule-associated protein 1 light chain 3 B (LC3B). More sustained mechanostimulation promotes fission and biogenesis of MDVs more effectively. Our results shed light on a mechanistically and functionally distinct type of fission driven by tensile forces, contributing to our understanding of the heterogeneity of fission and mechanoregulation of mitochondrial dynamics and MDV biogenesis. In addition, our results reveal the unique role of tail-autotomy fission in segregating only outer membrane components, indicating a distinct approach for mitochondrial quality control.

## Results

### Tail-Autotomy Fission Occurs After Mitochondrial Tubulation.

**Fig. 1.**
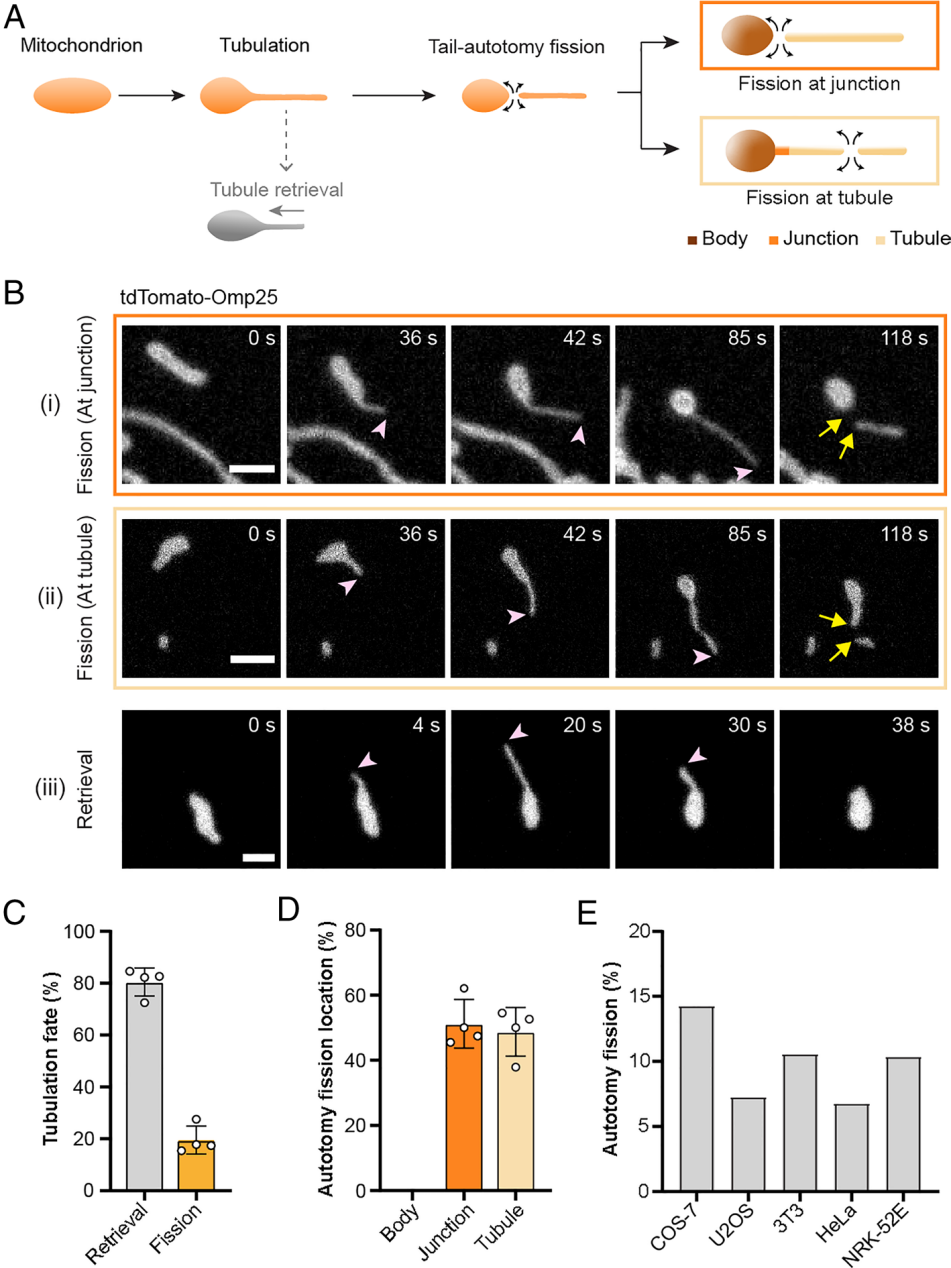
Tail-autotomy fission occurs after mitochondrial tubulation. (*A*) Schematic representation of tail-autotomy fission, wherein a tail-like thin tubule first protrudes from the mitochondrial body and next disconnects either at the body-tubule junction or at the tubule. The extending tubule can also retract back to the body instead of dividing. In (*B–D*), COS-7 cells were transfected with tdTomato-Omp25. (*B*) Representative images of tail-autotomy fission at tubule-body junction (i) or at tubule (ii) and the retrieval of tubule (iii). Pink arrowheads indicate extending tubules and yellow arrows mark fission. (*C*) Percentage of tail-autotomy fission or tubule retrieval after tubulation. Circles, individual values from four independent experiments, 601 events in total. (*D*) Percentage of fission events occurring at different submitochondrial sites. Circles, individual values from four independent experiments, 109 fission events in total. (*E*) Percentage of tail-autotomy fission in all spontaneous fission in COS-7, U2OS, 3T3, HeLa, and NRK-52E cells. >160 fission events quantified for each cell type. Data presents mean ± SD (*C* and *D*). (Scale bars, 2 μm.)

We inspected spontaneous mitochondrial dynamics without any pharmacological induction in COS-7 cells expressing tdTomato-Omp25. Omp25 encodes the transmembrane domain of Omp25 (outer membrane protein 25, a.a. 111 to 145) which anchors to OMM to target mitochondria (13). Frequent mitochondrial tubulation events were observed (Fig. 1 *A* and *B*), in good agreement with previous reports (10, 11, 14). It is worth noting that upon the outward projection of thin tubules, many mitochondria exhibited a typical body-tail morphology with a long thin tail-like tubule tethered to a round body (Fig. 1*A*), which can be, to a first approximation, understood from the energetics of its lipid membranes (15, 16). As the growing tubule drags more membrane from the body, the vesicle body becomes increasingly spherical due to volume conservation under a decreasing surface area (17). We continued to record the fates of the elongated tubules, but excluded from our analysis those tubules that reached and then fused with another mitochondrion. While some tubules were eventually retrieved back toward the body as described previously (10, 11), some underwent fission by being disconnected from the body, resembling the autotomy of the tail (Fig. 1 *A* and *B*), which is denoted as tail-autotomy fission thereafter. Analysis of hundreds of mitochondrial tubulations showed that 19.5% of tubules went through tail-autotomy fission rather than tubule retrieval (Fig. 1*C*). A closer look at the fission sites revealed that fission could take place either at body-tubule junctions or in the middle of the tubules, while none on the chubbier body. Further quantification indicates that autotomy fission occurs at the junction (52.2%) or within the tubules (48.8%) at similar frequencies (Fig. 1*D*). In COS-7 cells, 14.3% of all the naturally occurring fission is tail-autotomy fission (Fig. 1*E* and *SI Appendix*, Fig. S1*A* and Movie S1), and the ratios range from 6.8% to 10.6% in U2OS, 3T3, HeLa and NRK-52E (Fig. 1*E*).

### Mechanical Tensile Force Induces Tail-Autotomy Fission.

**Fig. 2.**
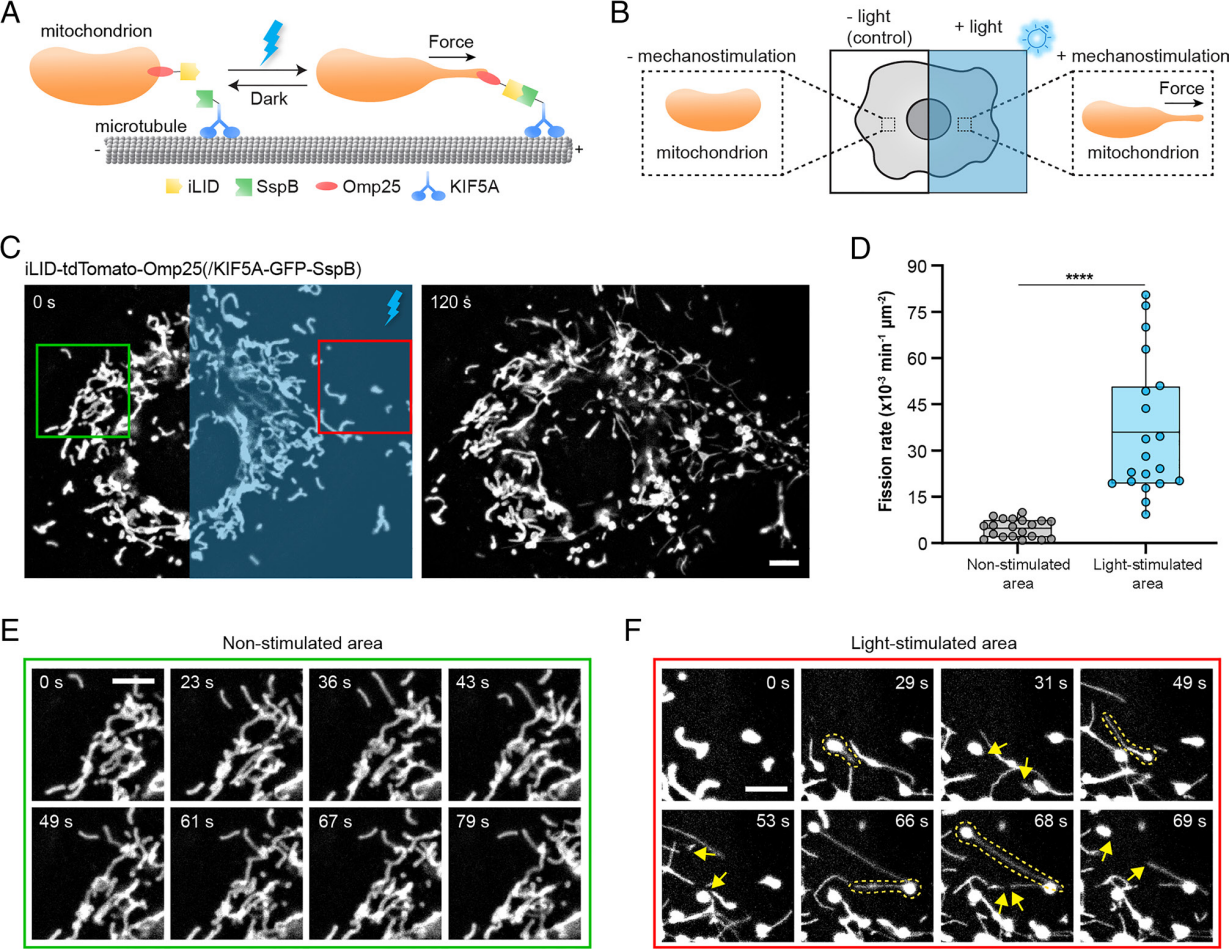
Mechanical tensile force by optogenetic mechanostimulator drives mitochondrial tubulation and subsequent tail-autotomy fission. (*A*) Schematic representation of light-gated mechanostimulation imposed on mitochondria. iLID is fused to Omp25 to target mitochondrial membrane, while SspB is fused to the kinesin motor protein encoded by KIF5A. Blue light-gated iLID/SspB dimerization induces the recruitment of kinesins to mitochondria, thus exerting pulling force on mitochondria. (*B*) Schematic representation of experimental design. Blue light is delivered to one side of the cell to apply mitochondria-specific mechanostimulation, and the other side is used as a nonstimulated control. (*C*) Fluorescence images of mitochondria in the COS-7 cell expressing iLID-tdTomato-Omp25 and KIF5A-GFP-SspB. The blue rectangle-marked area received intermittent blue light stimulation for 120 s. (*D*) Mitochondrial fission rates in the blue light-illuminated and nonilluminated areas of the same cell. N = 20 cells from seven independent experiments. Line, mean; bounds of box, 25th and 75th percentiles; whiskers, minimum to maximum value; circles, values in individual cells. ****P < 0.0001 using two-tailed paired Student’s *t* test. (*E*) Zoomed-in images of the region marked by the green box in the nonstimulated area in (*C*). (*F*) Zoomed-in images of the region marked by the red box in the blue light-stimulated area in (*C*). Yellow dashed lines outline mitochondria with force-induced tubulation which later underwent tail-autotomy fission (indicated by yellow arrows). (Scale bars, 5 μm.)

We hypothesized that mechanical pulling force is the key driver for tail-autotomy fission by piecing together three pivotal findings. First, governed by the physics of membrane deformation, it has been well established that a sufficiently large tensile force acting on a lipid vesicle can pull out a narrow tubule from the vesicle, producing a typical shape with a sphere tethered by a thin tubule (17, 18). Mitochondria preceding tail-autotomy fission exhibit the same morphological features (Fig. 1*B*). Second, previous reports have experimentally proven that pulling forces, provided either by microtubule-associated molecular motors or hitchhiked organelles (10, 11), can elicit mitochondrial tubulation both inside cells or in vitro. Third, it has been shown that higher mitochondrial membrane tension leads to higher frequency of fission (19). To further support this hypothesis, we found that after the disruption of microtubules by Nocodazole treatment, few spontaneous tubulations or tail-autotomy fission could be observed (*SI Appendix*, Fig. S1*B*). To test the hypothesis, we used our recently developed optogenetic strategy to exert pulling forces specifically toward intracellular mitochondria (12). In our design, the optical dimerizer iLID (improved light-induced dimer) and the microvariant of SspB were utilized which bind together within seconds after blue light exposure (20). iLID was fused with tdTomato and Omp25 to target and fluorescently mark mitochondria (Fig. 2*A*). A truncated version of KIF5A which is unable to bind to cargos was fused with SspB (21). Upon blue light illumination, iLID/SspB interaction leads to the recruitment of kinesins to OMM, thus imposing pulling forces generated by kinesins on mitochondria (*SI Appendix*, Fig. S2).

To probe the causal linkage between tensile forces and tail-autotomy fission, we took advantage of the spatial precision offered by the optogenetic mechanostimulator and optically exerted forces on mitochondria residing within one side of the cell, while mitochondria on the other nonilluminated side remained unperturbed as a control (Fig. 2*B*). COS-7 cells were transfected with iLID-tdTomato-Omp25 and KIF5A-GFP-SspB. As shown in Fig. 2*C* and Movie S2, the morphology and dynamics of mitochondria within the nonilluminated area remained largely unaltered. Contrastingly, in the blue light-illuminated region marked by a blue rectangle, the mitochondrial network became more fragmented as a result of active mitochondrial fission. Close examination of the nonstimulated area marked by the green box showed no noticeable deformation of mitochondria or fission (Fig. 2*E*). However, in the stimulated area indicated by the red box, thin and long mitochondrial tubules were pulled out which later underwent tail-autotomy fission as indicated by the yellow arrows (Fig. 2*F*). More examples of tail-autotomy fission driven by light-gated tensile force can be found in *SI Appendix*, Fig. S1*C*. Quantification of fission rates in the stimulated and unstimulated areas confirmed the significantly increased level of mitochondrial fission in the light-stimulated area (Fig. 2*D*). Therefore, our results show that mechanical tensile force can directly promote tail-autotomy fission.

### Sustained Force Promotes Tail-Autotomy Fission More Efficiently Than Transient Force.

**Fig. 3.**
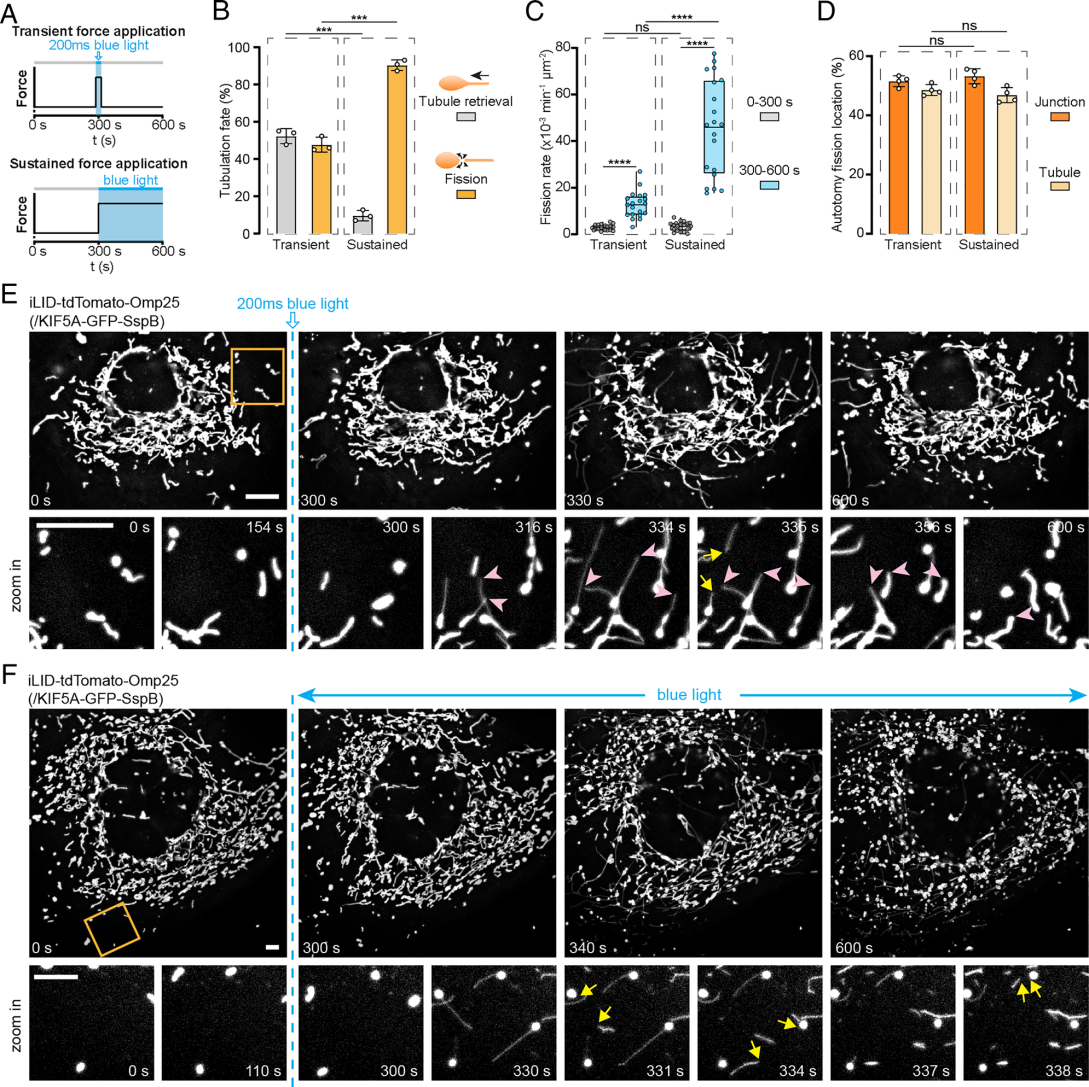
Sustained force promotes tail-autotomy fission more efficiently than transient force. (*A*) Illustrative scheme for transient or sustained force application by blue light. One pulse of 200 ms blue light is delivered for transient mechanostimulation and intermittent blue light exposure for sustained mechanostimulation. (*B*) Percentage of protruding mitochondrial tubules undergoing fission or retrieval after blue light-mediated transient or sustained mechanostimulation. N ≥ 409 force-induced tubulation events from 10 cells in three independent experiments per group. (*C*) Quantification of fission rates in the same cell before (0 to 300 s) and after (300 to 600 s) the onset of light stimulation. N = 20 cells from 6 independent experiments per group. Line, mean; bounds of box, 25th and 75th percentiles; whiskers, minimum to maximum value; circles, values in individual cells. ns (not significant) *P* > 0.05, ****P < 0.0001 using two-tailed paired Student’s *t* test for the same condition, and two-tailed unpaired Student’s *t* test for different conditions. (*D*) Percentage of fission events occurring at different sites upon transient or sustained force stimulation. N ≥ 150 fission events from more than 12 cells in four independent experiments. In (*B*) and (*D*), data present mean ± SD; circles, values in individual experiments; ns, *P* > 0.05, ***P < 0.001 using two-tailed unpaired Student’s *t* test. (*E*) Fluorescence images of mitochondria before and after transient light stimulation. Zoomed-in images of the yellow box-marked area showed many mitochondrial tubulations, followed by retrieval of the tubules (indicated by pink arrowheads), or tail-autotomy fission (marked by yellow arrows). (*F*) Fluorescence images of mitochondria before and after sustained light stimulation. Zoomed-in images of the yellow box-marked area showed elongation of the three mitochondria and subsequent tail-autotomy fission (marked by yellow arrows). COS-7 cells were transfected with iLID-tdTomato-Omp25 and KIF5A-GFP-SspB. (Scale bars, 10 μm.)

Next, we found that sustained force application promotes more tail-autotomy fission than transient one. After the extension of the thin tubule via exertion of tensile force, the fates of tubules that didn’t fuse with other mitochondria diverged by either disconnecting from or retrieving back toward the body (Fig. 1*B* and *SI Appendix*, Fig. S1*C*). We hypothesized that the duration of force affects the fates of tubules. To probe this hypothesis, we leveraged the temporal precision of our optogenetic method to apply transient or sustained pulling forces to intracellular mitochondria via varied light stimulation durations. Such precise temporal controllability relies on the reversible light-gated association between iLID and SspB so that blue light removal abrogates the light-mediated force application. In COS-7 cells expressing iLID-tdTomato-Omp25 and KIF5A-GFP-SspB, to impose transient mechanical force, one pulse of 200 ms blue light was delivered, while intermittent blue light exposure for 5 min was adopted for more sustained mechanostimulation (Fig. 3*A*). To measure the effects of light-gated mechanostimulation, mitochondria dynamics were monitored 5 min before and after the onset of blue light exposure.

As shown in Fig. 3*E* and Movie S3, a transient pulling force induced tubule elongation and some mitochondrial fission events, which did not cause significant fragmentation of the entire mitochondrial network. The zoomed-in examination of the area highlighted by the yellow box showed that after the force-triggered mitochondrial tubulation, many tubules were withdrawn toward the mitochondrial body (indicated by pink arrowheads). Only one underwent fission, as marked by the yellow arrows. On the contrary, sustained mechanostimulation induced dramatic mitochondrial deformation and frequent fission, which resulted in noticeable mitochondrial fragmentation (Fig. 3*F* and Movie S4). As shown in the zoomed-in area, mitochondria remained mostly unchanged before light stimulation. Contrastingly, blue light exposure drove the protrusion of mitochondrial tubules and the subsequent tail-autotomy fission of the three mitochondria indicated by the yellow arrows. Compared to transient force, sustained force application leads to a higher proportion of tubule-protruding mitochondria undergoing fission rather than tubule retrieval (Fig. 3*B*). We calculated the mitochondrial fission rates in the same cell before (t = 0 to 300 s) and after (t = 300 to 600 s) the start of blue light stimulation. Quantification indicates that although both transient and sustained mechanostimulation increased the rates of fission, sustained force application is much more efficient in promoting fission (Fig. 3*C*). This may be due to that longer force application can pull out longer tubules and thus heighten the membrane tension. It can also maintain the tubular structures and prevent their retrieval, gaining more time for fission machinery to get recruited on tubules. In addition, we found that durations of force exertion did not affect the fission sites. Either sustained or transient mechanostimulation yielded similar rates of fission at body-tubule junction or within tubules (Fig. 3*D*).

We further demonstrated that tail-autotomy fission can be elicited by the optogenetic mechanostimulator utilizing another mitochondrial targeting sequence (mitochondrial Rho GTPase 1, Miro1), other optical heterodimerizing pairs (CRY2/CIBN and LOVpep/ePDZ), and other motor proteins (kinesin 3 motor KIF1A, a.a. 1-383), and in U2OS cells (*SI Appendix*, Fig. S3). Thus, our results show that tail-autotomy fission by light-gated tensile force is independent of any components in our optogenetic system and can be achieved in different types of cells.

### Force-Induced Tail-Autotomy Fission Depends on DRP1 and MFF.

**Fig. 4.**
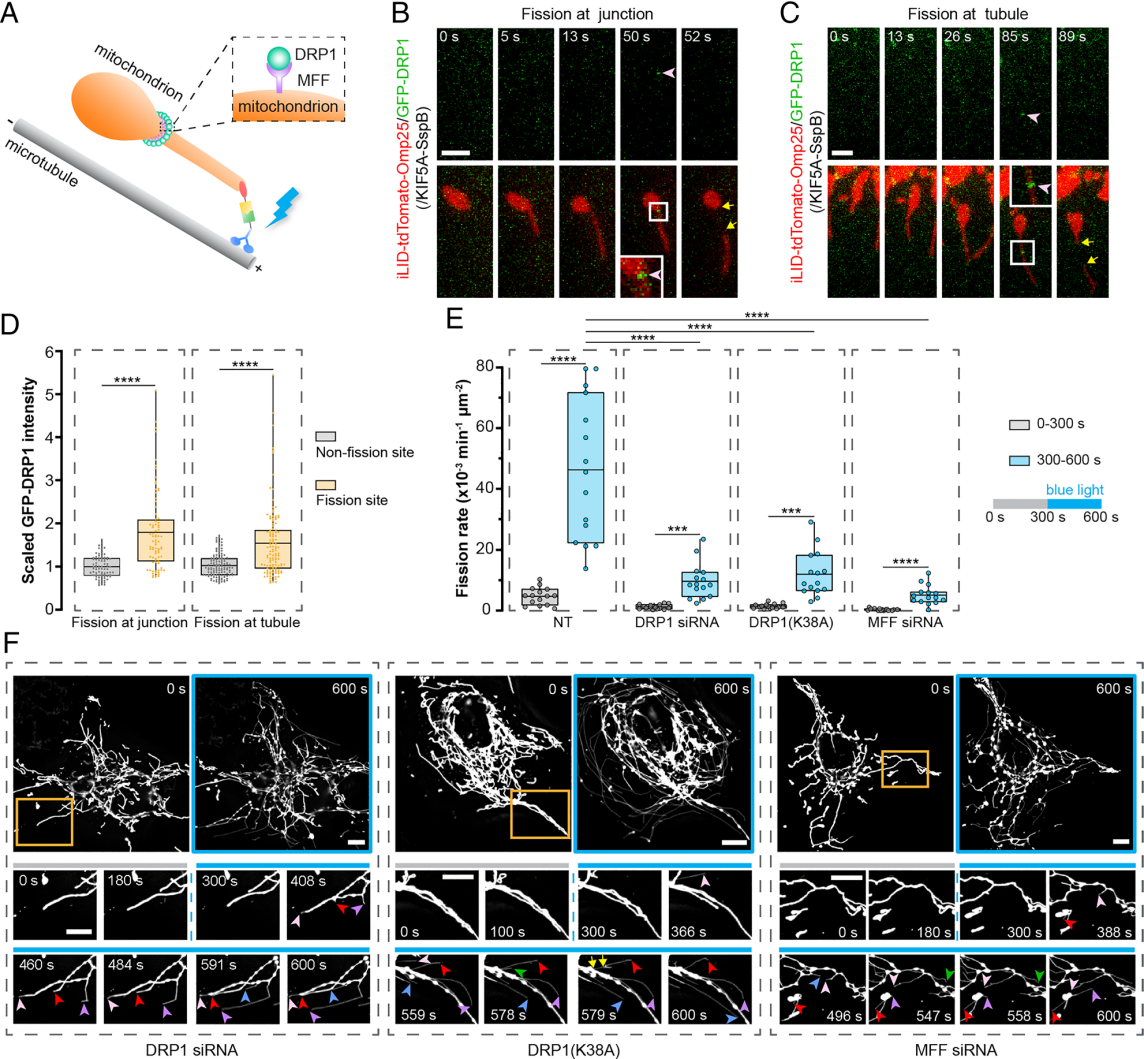
Force-induced tail-autotomy fission depends on DRP1 and MFF. (*A*) Illustration scheme showing the involvement of MFF and DRP1 at force-induced mitochondrial fission sites. (*B* and *C*) The formation of DPR1 puncta at the force-induced fission sites. COS-7 cells were transfected with iLID-tdTomato-Omp25, KIF5A-SspB, and GFP-DRP1. DRP1 puncta (pointed by the pink arrowhead) was formed at the fission site at the junction (*B*) or at the tubule (*C*). (*D*) Quantification of DRP1 intensity at the nonfission and fission sites on the same mitochondrion. N ≥ 80 fission events per group. Statistical analysis using Wilcoxon matched-pairs signed ranks test. In (*E* and *F*), to suppress DRP1 activities in COS-7 cells transfected with iLID-tdTomato-Omp25 and KIF5A-SspB, one group was treated with DRP1-targeted siRNA and another group with overexpression of DRP1(K38A). MFF-targeted siRNA was utilized to knock down MFF. (*E*) Mitochondrial fission rates in the nontreated control group (NT), DRP1 siRNA treated group, DRP1(K38A)-expressing group, and MFF siRNA treated group. N = 15 cells from ≥4 independent experiments per group. Statistical analysis using two-tailed paired Student’s *t* test for the same condition, and two-tailed unpaired Student’s *t* test for different conditions. In (*D*) and (*E*), line, mean; bounds of box, 25th and 75th percentiles; whiskers, minimum to maximum value; circles, values in individual fission events (*D*) or cells (*E*). ***P < 0.001, ****P < 0.0001. (*F*) Fluorescence images of mitochondria in cells treated with DRP1 siRNA, DRP1(K38A) overexpression, or MFF siRNA before and after blue light stimulation. Many mitochondria underwent force-induced tubulation (indicated by arrowheads), while few fission events occurred (indicated by yellow arrows). [Scale bars, 2 μm (*B* and *C*) and 10 μm (*F*).]

Here, we investigated the involvement of dynamin-related protein 1 (DRP1) and mitochondrial fission factor (MFF) in force-induced mitochondrial fission. As the master regulator for mitochondrial fission, DRP1 is recruited to the mitochondrial surface, forming an oligomeric ring to drive fission (22). MFF is a type of DRP1 receptor to recruit DRP1 to OMM (23, 24). First, to explore the participation of DRP1, we transfected COS-7 cells with GFP-DRP1 as well as the optogenetic constructs (iLID-tdTomato-Omp25 and KIF5A-SspB) (Fig. 4*A*). After blue light illumination, we found that shortly after DRP1 foci were formed on the mitochondrial tubule or junction (Fig. 4 *B* and *C*, and more examples in *SI Appendix*, Fig. S4 *A* and *B*), mitochondria were divided at these sites and then the DRP1 foci soon disappeared. Quantification of GFP-DRP1 intensity on mitochondria confirmed the enrichment of DRP1 on fission sites compared to nonfission sites (Fig. 4*D*), indicating the presence of DRP1 in force-induced fission.

To further probe the involvement of DRP1 and MFF, we checked whether and how DRP1 or MFF deficiency can influence force-induced fission. First, the activity of DRP1 in COS-7 cells was inhibited by the treatment of DRP1-targeted siRNA to knockdown DRP1 (*SI Appendix*, Fig. S4*C*), or the overexpression of a dominant negative DRP1 mutant, DRP1(K38A) (25), which interacts with and consequently inhibits the GTPase activity of wildtype DRP1 (26, 27). The activity of MFF was inhibited by the treatment of MFF-targeted siRNA (*SI Appendix*, Fig. S4*C*). Inhibition of DRP1 and MFF generated many hyperfused mitochondria (Fig. 4*F*), indicating the successful suppression of DRP1 or MFF functions. Next, we compared the fission rates before and during the 5 min intermittent blue light stimulation in the same cells. Blue light still triggered the drastic extension of thin mitochondrial tubules in cells (Fig. 4*F*). However, few mitochondria divided following the force-induced mitochondrial elongation. Quantification of fission rates showed that DRP1(K38A) overexpression (12.7 × 10^−3^ min^−1^ μm^−2^), DRP1 knockdown (8.7 × 10^−3^ min^−1^ μm^−2^), or MFF knockdown (4.5 × 10^−3^ min^−1^ μm^−2^) significantly lowered the frequency of force-triggered fission compared to the control group (46.2 × 10^−3^ min^−1^ μm^−2^) (Fig. 4*E*). To exclude the possibility that the long length of mitochondria is the key factor suppressing fission, we demonstrated that long tubular mitochondria in COS-7 cells with functional DRP1 and MFF could still undergo frequent force-triggered fission (*SI Appendix*, Fig. S4*D*). Therefore, our results indicate that both MFF and DRP1 participate in tensile force-induced tail-autotomy fission.

### ER Tubules Cross Over at Force-Induced Mitochondrial Fission Sites.

**Fig. 5.**
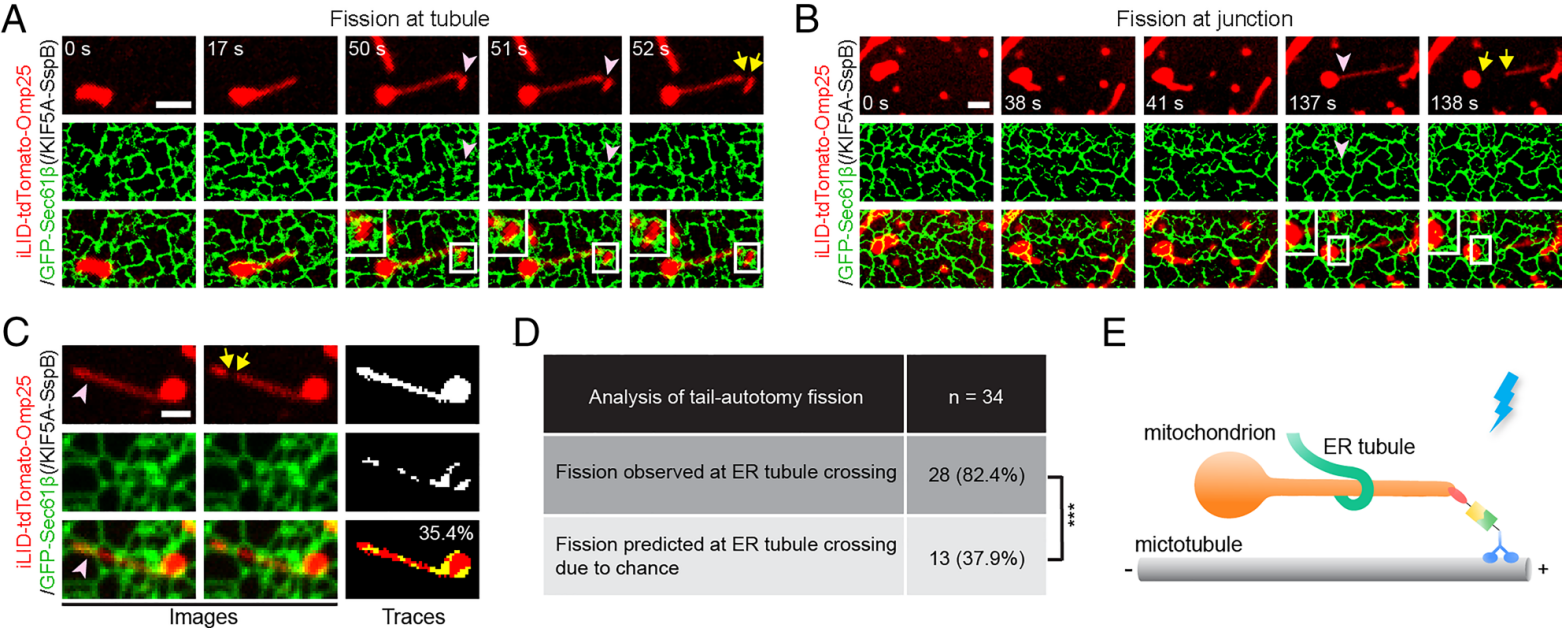
ER tubules cross over at force-induced mitochondrial fission sites. (*A* and *B*) Fluorescence images of ER and mitochondria. ER tubules wrapped the mitochondria at mitochondrial fission sites (indicated by pink arrowheads), either on the extending tubule (*A*) or at the body-tubule junction (*B*). Fission events are indicated by yellow arrows. (*C*) An example to show the method for determining the percentage of mitochondrial surface covered by ER tubule crossing from an image captured just prior to tail-autotomy fission. The left two columns are fluorescence images. The right column shows tracing with the percentage of mitochondrial pixels colocalized with pixels from ER tubules crossing the mitochondrion. (*D*) The actual number of fission events at ER-mitochondrial contact sites compared to the expected number of fission at ER contact if there is no relationship between ER and mitochondrial division (predicted from the percentage of the mitochondria surface covered by crossing ER tubules for 34 tail-autotomy fission). ***P < 0.001, Fisher’s exact test. (*E*) Schematic illustration of ER tubules wrapping around the extending mitochondrial tubules to mediate force-induced fission. COS-7 cells were transfected with iLID-tdTomato-Omp25, KIF5A-SspB, and GFP-Sec61β. [Scale bars, 5 μm (*A* and *B*) and 2 μm (*C*).]

ER has been identified as an important player in mitochondrial fission by utilizing ER tubules to wrap around and mediate the fission of mitochondria (28). Here, we examined whether ER tubules were present at mitochondrial fission sites upon forced-induced tail-autotomy division. COS-7 cells were transfected with iLID-tdTomato-Omp25 and KIF5A-SspB as well as GFP-Sec61β that marks the ER structures via the transmembrane domain of Sec61β, the β subunit of Sec61 complex localized on ER (29). Upon blue light-mediated mitochondrial elongation, the extending mitochondria tubule reached the network of ER. At the sites of fission either on the tubule (Fig. 5*A*) or at the body-tubule junctions (Fig. 5*B*), ER wrapped around the mitochondria right before the fission occurred. We adopted a previously established method (28) and found that the frequency of ER-associated mitochondrial division (82.4%) was much higher than that predicted by the percentage of mitochondrial areas covered by crossing ER tubules (37.9%) (Fig. 5 *C* and *D*). If ER is randomly distributed over the site of force-induced fission, then the two numbers should be similar. Our results indicate that ER indeed actively participates in force-triggered mitochondrial fission (Fig. 5*E*), echoing the critical role of ER in mitochondrial fission found in many other studies (30, 31).

### Force-Induced Tail-Autotomy Fission Generates Mitochondrial Fragments with or without mtDNA for Different Fates.

**Fig. 6.**
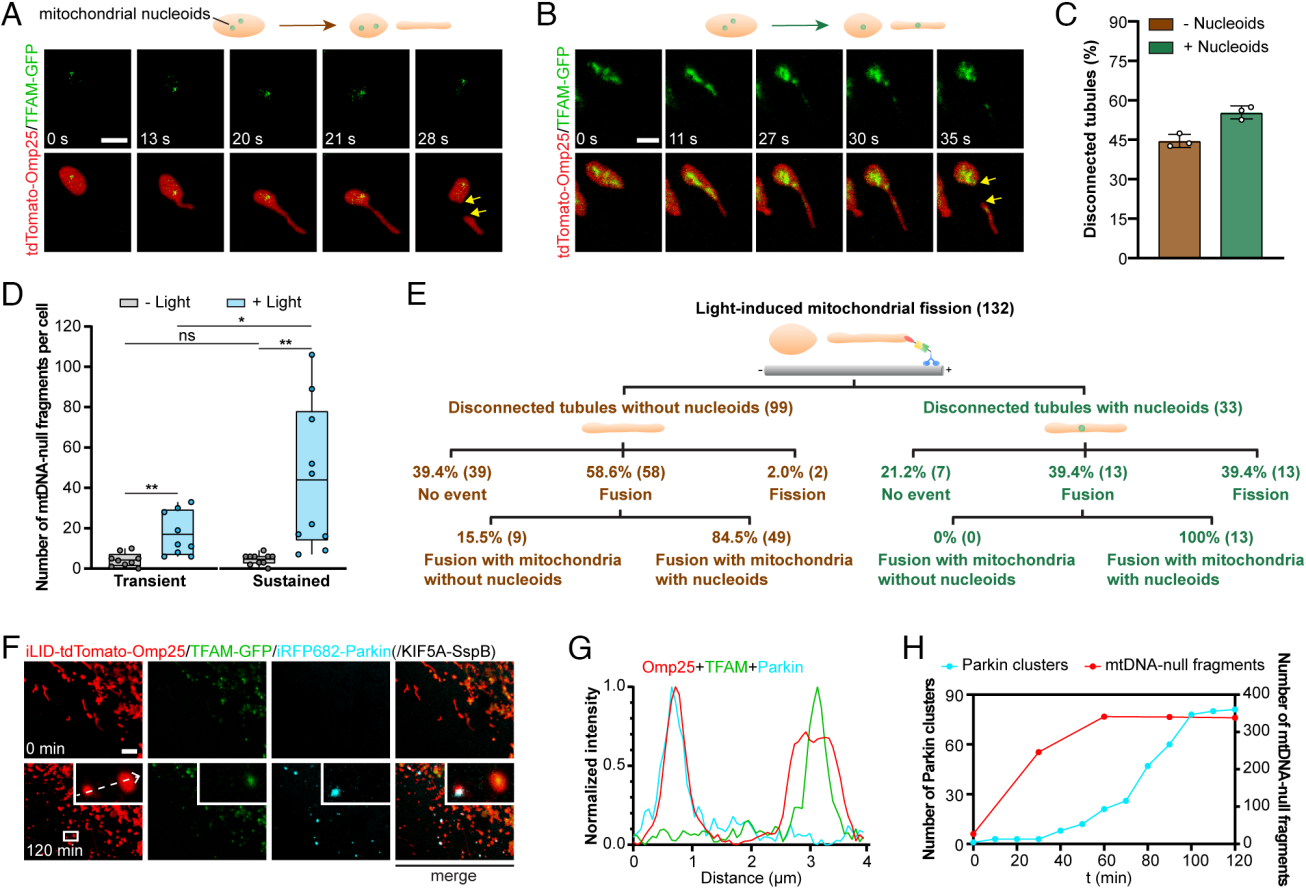
Force-induced tail-autotomy fission generates mitochondrial fragments with or without mtDNA for different fates. (*A* and *B*) Fluorescence images of mitochondria and mtDNA during naturally occurring tail-autotomy fission. mtDNA stayed in the mitochondrial body (*A*) or got redistributed into the disconnected tubule (*B*). (*C*) Percentage of tail-autotomy fission with disconnected tubules containing or not containing mtDNA. N = 107 fission events from three independent experiments. Circles, values of individual experiments. Data presents mean ± SD. (*D*) The numbers of mtDNA-null fragments in cells before light stimulation, and 1 min after one 200 ms pulse light stimulation (transient force) or 1 min intermittent blue light stimulation (sustained force). N ≥ 9 cells per group. Line, mean; bounds of box, 25th and 75th percentiles; whiskers, minimum to maximum value; circles, values of individual cells. Statistical analysis using two-tailed paired Student’s *t* test for the same group, and two-tailed unpaired Student’s *t* test for different groups. ns, *P* > 0.05, *P < 0.05, **P < 0.01. (*E*) Schema depicting the fates of the disconnected tubule fragments which were tracked for 300 s after force-induced fission. (*F*) Fluorescence images of mitochondria, mtDNA, and Parkin after intermittent blue light stimulation for 30 min and examined for 120 min. Tubule fragments without mtDNA recruited Parkin proteins. (*G*) Plot profiles showing the fluorescence intensity of iLID-tdTomato-Omp25, TFAM-GFP, and iRFP682-Parkin along the white dashed arrow in (*F*). (*H*) The numbers of Parkin puncta and mtDNA-null fragments over time in the cell in (*F*). In (*A–C*) COS-7 cells were transfected with tdTomato-Omp25 and TFAM-GFP. In (*D–H*), COS-7 cells were transfected with iLID-tdTomato-Omp25, TFAM-GFP and KIF5A-SspB (*D* and *E*) or along with iRFP682-Parkin (*F–H*). [Scale bars, 2 μm (*A* and *B*) and 5 μm (*F*).]

Inspired by a recent finding that mitochondrial tubulation can drive the active transportation of mtDNA into tubule segments (14), we set out to investigate the allocation of mtDNA into daughter vesicles via tail-autotomy fission. Residing inside the mitochondrial matrix, mtDNA is the small and circular DNA essential for mitochondrial functions (32). To label mtDNA in living cells, TFAM, a mitochondrial transcription factor that packages mtDNA into nucleoids (33), was fused with GFP. First, in COS-7 cells expressing TFAM-GFP and tdTomato-Omp25, we observed that spontaneous tail-autotomy fission induced asymmetrical segregation of mtDNA, keeping the majority of mtDNA inside the body fragments and generating tubule fragments with (55.4%) or without (44.6%) mtDNA (Fig. 6 *A*–*C*, and more examples in *SI Appendix*, Fig. S5 *A* and *B*). Next, we utilized the optogenetic mechanostimulator to probe the determinants and outcomes of such mtDNA redistribution. COS-7 cells were transfected with iLID-tdTomato-Omp25, KIF5A-SspB, and TFAM-GFP. Sustained force application by 1 min intermittent blue light stimulation generated more mtDNA-null tubule fragments than transient force exertion by one 200 ms pulse light stimulation (Fig. 6*D*, and examples in *SI Appendix*, Fig. S5 *C–F*). Furthermore, we examined the fates of these disconnected mitochondrial fragments with or without mtDNA 300 s after force-triggered fission (Fig. 6*E*, and examples in *SI Appendix*, Fig. S6). For those with mtDNA, 39.4% of them could still undergo fission. Another 39.4% of them later fused with another mitochondrion containing mtDNA. On the contrary, for disconnected mitochondrial fragments without mtDNA, only 2% were divided afterward. Of note, 58.6% of them went through fusion. Intriguingly, most of them fused with mitochondria with mtDNA (84.5%), possibly to provide mtDNA complementation for further repair.

We proceeded to examine the fates of mtDNA-null mitochondrial fragments which failed to fuse with another mitochondrion containing mtDNA. PINK1/Parkin are core organizers in eliminating damaged mitochondria for mitochondrial quality control (34, 35). Parkin amplifies a mitochondrial damage detection signal from PINK1 and recruits more Parkin to mitochondria (36, 37). We found that Parkin could sense and mark mtDNA-null mitochondrial fragments generated by tail-autotomy fission. Cells were transfected with iLID-tdTomato-Omp25, TFAM-GFP, KIF5A-SspB, and iRFP682-Parkin and subjected to intermittent blue light illumination for 30 min. Around 40 min after the start of light stimulation, discernible Parkin clusters formed exclusively on mtDNA-null mitochondria and increased over time (Fig. 6*F*, whole cell images at different time points and quantification in *SI Appendix*, Fig. S7). The examination of the fluorescence intensity along the white dashed arrow confirmed the colocalization of Parkin clusters and mtDNA-null fragments (Fig. 6*G*). We quantified the number of Parkin clusters at different time points and confirmed its gradual increase along with the formation of mtDNA-null fragments over time (Fig. 6*H*).

### Tensile Force Induces OMM/IMM Separation and the Formation of Matrix-Excluded MDVs which Recruit Parkin and LC3B.

**Fig. 7.**
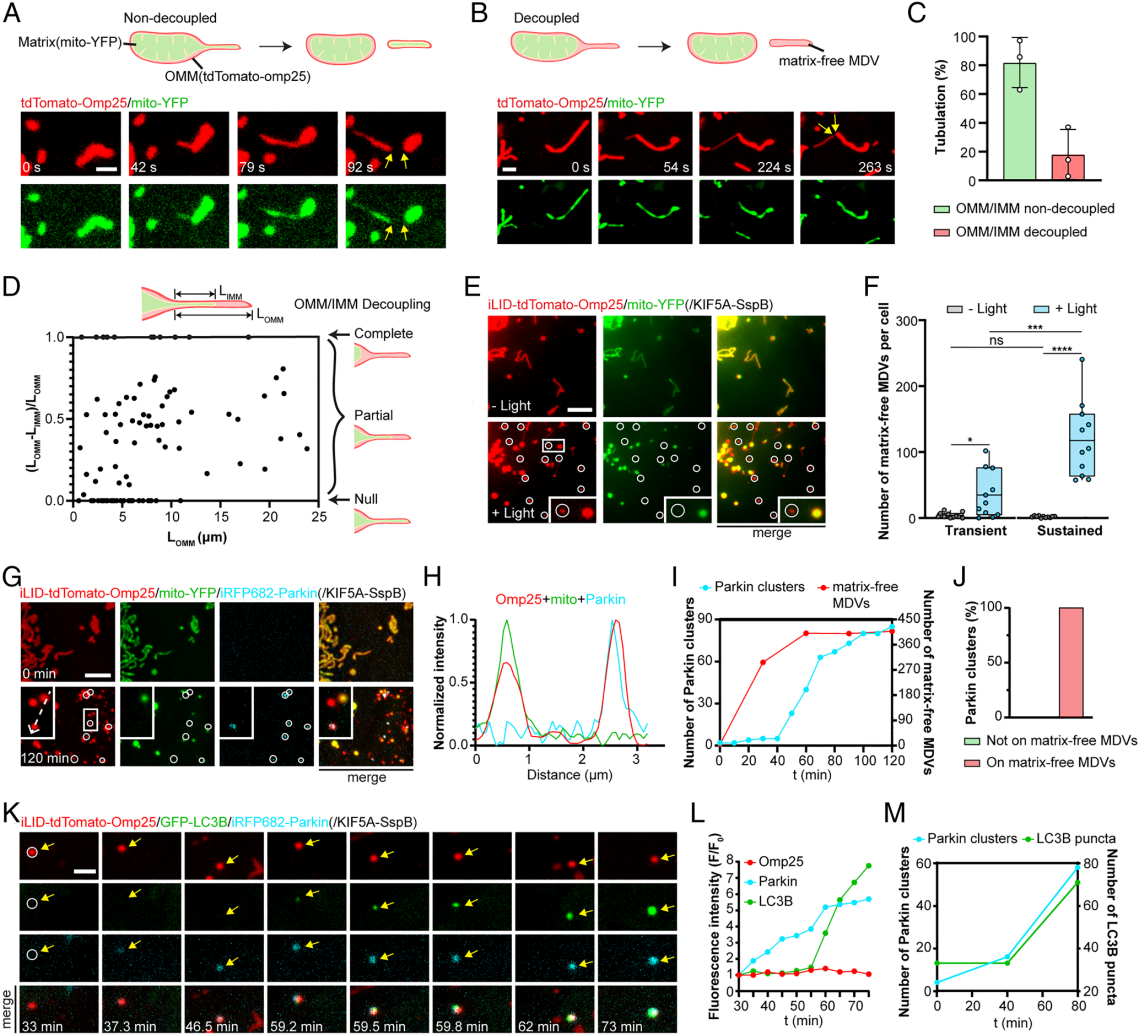
Tensile force induces OMM/IMM separation and the formation of matrix-excluded MDVs which recruit Parkin and LC3B proteins. In (*A–C*) COS-7 cells were transfected with tdTomato-Omp25 and mito-YFP. (*A* and *B*) Fluorescence images of mitochondria undergoing spontaneous tubulation without (*A*) or with (*B*) OMM/IMM decoupling, dividing tubule fragments with (*A*) or without (*B*) mitochondrial matrix by subsequent tail-autotomy fission. Matrix-excluded tubule fragments generated are denoted as matrix-free MDVs. (*C*) Percentage of spontaneous mitochondrial tubulation with or without OMM/IMM decoupling. N = 159 events from three independent experiments. Circles, values of individual experiments. Data presents mean ± SD. In (*D–J*), COS-7 cells were transfected with iLID-tdTomato-Omp25, mito-YFP, and KIF5A-SspB (*D–F*) as well as iRFP682-Parkin (*G–J*). (*D*) Analysis of OMM/IMM decoupling upon different lengths of mitochondrial tubulation. N = 104 tubulation events. (*E*) Fluorescence images of OMM and mitochondrial matrix to show the formation of matrix-free MDVs after light stimulation. (*F*) The numbers of matrix-free MDVs in cells before light stimulation, and 10 min after one 200 ms pulse light stimulation (transient force) or 10 min intermittent blue light stimulation (sustained force). N = 11 cells per group. Line, mean; bounds of box, 25th and 75th percentiles; whiskers, minimum to maximum value; circles, values of individual cells. Statistical analysis using two-tailed paired Student’s *t* test for the same group, and two-tailed unpaired Student’s *t* test for different groups. ns, *P* > 0.05, *P < 0.05, ***P < 0.001, ****P < 0.0001. (*G*) Fluorescence images of mitochondria, mitochondrial matrix, and Parkin after intermittent light stimulation for 30 min. Matrix-free MDVs recruited Parkin proteins (indicated by white circles). (*H*) Plot profiles showing the fluorescence intensity of iLID-tdTomato-Omp25, mito-YFP, and iRFP682-Parkin along the white dashed arrow in (*G*). (*I*) The number of Parkin puncta and matrix-free MDVs over time in the cell in (*G*). (*J*) Percentage of Parkin clusters colocalized on matrix-excluded fragments. N = 140 Parkin clusters from three independent experiments. In (*K–M*), COS-7 cells were transfected with iLID-tdTomato-Omp25, GFP-LC3B, iRFP682-Parkin and KIF5A-SspB. (*K*) LC3B was recruited to the mitochondrial fragment colocalized with the Parkin cluster. (*L*) Fluorescence intensity of iLID-tdTomato-Omp25, GFP-LC3B, and iRFP682-Parkin on the matrix-free MDV in (*K*). (*M*) The number of Parkin clusters and LC3B puncta over time in the cell in (*K*). [Scale bars, 2 μm (*A*, *B,* and *K*) and 5 μm (*E* and *G*).]

We further asked how the mitochondrial matrix is redistributed during tail-autotomy fission, as mtDNA only constitutes part of the matrix. We revealed that mitochondrial tubulation can protrude tubules containing only OMM but no matrix. Subsequent tail-autotomy fission shed the tubule fragments into matrix-excluded MDVs which later recruit Parkin and LC3B. MDVs are small membranous vesicles released by mitochondria that are recognized as critical contributors to mitochondrial quality control (38–40) and interorganelle communication (40–42). The formation of MDVs containing no mitochondrial matrix, denoted as matrix-free MDVs hereafter, has been previously reported by different groups (41, 43, 44). To start with, we showed that spontaneous tail-autotomy fission generated matrix-free MDVs. COS-7 cells were transfected with tdTomato-Omp25 as well as mito-YFP to label the mitochondrial matrix and the boundary of IMM. Many mitochondria underwent simultaneous tubulation of both IMM and OMM (81.05%) (*SI Appendix*, Fig. S8*A*). Contrastingly, some mitochondrial tubulation decoupled IMM and OMM (18.95%), creating tubule segments void of mitochondrial matrix (Fig. 7*C* and *SI Appendix*, Fig. S8 *B* and *C*). Therefore, ensuing tail-autotomy fission generated tubule fragments with matrix or matrix-free MDVs (Fig. 7 *A* and *B*, more examples in *SI Appendix*, Fig. S8 *D* and *E*). The biogenesis of matrix-free MDVs by tail-autotomy fission is not surprising because both tubulation without mitochondrial matrix (12) and tubulation-mediated formation of MDVs have been previously reported (45).

Then we used the optogenetic mechanostimulator to further investigate the force-induced biogenesis of matrix-free MDVs. COS-7 cells were transfected with iLID-tdTomato-Omp25, mito-YFP, KIF5A-SspB. First, how the lengths of OMM tubulation affect OMM/IMM separation was examined by quantifying the difference in OMM and IMM lengths in the tubule regions (Fig. 7*D*). The concurrent tubulation of OMM and IMM cannot be observed when tubulation was beyond 12 μm, indicating that longer tubulation promotes OMM/IMM decoupling. Next, we validated that tail-autotomy fission driven by tensile force indeed produced matrix-free MDVs in COS-7 cells (Fig. 7*E*, whole cell images in *SI Appendix*, Fig. S9*A*, and examples in *SI Appendix*, Fig. S8 *F–I*). Moreover, compared with transient force exerted by one pulse of 200 ms blue light, longer mechanostimulation by 10 min intermittent blue light exposure enhanced the biogenesis of matrix-free MDVs (Fig. 7*F*).

We proceeded to study the fates of matrix-free MDVs generated by tail-autotomy fission. We found that Parkin clusters formed exclusively on matrix-free MDVs, which later recruited LC3B. Cells were transfected with iLID-tdTomato-Omp25, mito-YFP, KIF5A-micro, and iRFP682-Parkin and subjected to intermittent blue light illumination for 30 min. Discernible Parkin clusters formed on mitochondrial fragments containing no mito-YFP signals (Fig. 7*G*, whole cell images in *SI Appendix*, Fig. S9*C*), as also shown by the examination of the fluorescence intensity (Fig. 7*H*). Those Parkin clusters gradually appeared after the rise of the total number of matrix-free MDVs (Fig. 7*I*). Moreover, all the Parkin clusters resided on matrix-free MDVs (Fig. 7*J*). Indeed, matrix-free MDVs inevitably contained no mtDNA, further explaining the fates of some mtDNA-null mitochondrial fragments shown in Fig. 6. We next assessed whether matrix-free MDVs with Parkin accumulation proceeded toward mitophagy. Colocalization of LC3 puncta and mitochondria has been widely used to demonstrate the presence of mitophagy (46–48). Here, we examined the distribution of GFP-LC3B following Parkin recruitment. In COS-7 cells expressing iLID-tdTomato-Omp25, KIF5A-SspB, GFP-LC3B, and iRFP682-Parkin, LC3B signal gradually accumulated around Parkin-enriched mitochondria (Fig. 7 *K*–*L*, whole cell images in *SI Appendix*, Fig. S9*B*). Quantification of the numbers of Parkin clusters and LC3B puncta in the cell confirmed the formation of LC3B puncta after the increase of Parkin clusters (Fig. 7*M*). Moreover, we validated that new LC3B puncta formed exclusively on matrix-free MDVs (*SI Appendix*, Fig. S9 *D* and *E*). Therefore, our results imply that via the biogenesis of matrix-free MDVs, tail-autotomy fission can serve as a unique mechanism to remove unwanted OMM components without discarding IMM or matrix, contributing to the various strategies for mitochondrial quality control.

## Discussion

In this study, we identified a distinct type of spontaneous fission, tail-autotomy fission, which is initiated by mechanical tensile force and features two dynamic steps, the extension of tail-like thin tubules from the mitochondrial body and the disconnection of the tubule fragments. The tail-autotomy fission accounts for a noticeable portion of all naturally occurring fission in multiple types of cells. Using an optogenetic mitochondria-specific intracellular mechanostimulator, we validated that tensile force can drive tail-autotomy fission, with sustained force exertion being more effective than transient one. Moreover, we revealed that such autotomy-like division leads to the asymmetrical segregation of mtDNA into daughter vesicles for different fates. It also generates MDVs without mitochondrial matrix which recruit Parkin and LC3B. Our findings enhance our understanding of the heterogeneity of mitochondrial fission, the direct mechanoregulation of mitochondrial fission and the biogenesis of MDVs, and assorted pathways for mitochondrial quality control.

The identification of tail-autotomy fission strengthens the emerging notion that mitochondrial fission is actually heterogeneous. The previously reported two types of fission, peripheral fission and midzone fission, are defined by different submitochondrial locations of fission and lead to differential mitochondrial fates (2). Here tail-autotomy fission is unique due to the initiatory and essential role of tensile force, characteristic morphological alterations, and its function for quality control. Mitochondria execute a spectrum of vital functions, and by themselves can display a wide diversity in size, morphology, and content (49, 50). Therefore, it is entirely conceivable that distinct types of fission are orchestrated to accurately match mitochondrial functions to cellular or subcellular needs varying constantly in space and time. As dysregulated fission has been implicated in diverse diseases, the heterogeneity of mitochondrial fission may necessitate precise therapeutic intervention that targets specific subpopulations of fission. Whether there are any other types of fission and what are their mechanisms and functional consequences await more investigations.

The tail-autotomy mitochondrial division always occurred at the protruding thin tubules instead of the bulkier body, probably due to the higher curvature-induced local membrane tension resulting from tubules’ smaller diameters. This is because higher mitochondrial membrane tension has been shown to promote mitochondrial division (19). Moreover, the protrusion of long mitochondrial tubules may also increase the likelihood of reaching adjacent ER structures and generating more ER-mitochondria contact sites to promote fission. On the other hand, our results expand the functional roles of mitochondrial tubulation. In addition to facilitating the formation of the mitochondrial network (10) and the transportation of mtDNA (14), here, we showed that mitochondrial tubulation directly mediates fission. Furthermore, our results present tensile force as a participant on the intricate map governing mitochondrial fission. Cells can exploit naturally occurring tensile forces, such as those provided by molecular motors, to actively modulate tail-autotomy mitochondrial fission and its quality control functions. More investigations are needed to unravel how the fission machinery senses or gets recruited onto the elongated thin tubules to trigger fission.

Upon the exertion of mechanical force, some mitochondria can be deformed while being repositioned, as shown in Figs. 2 and 3. Indeed, previous reports have shown that force provided by light-inducible motor recruitment can drive the intracellular transport of various organelles, such as lysosomes, peroxisomes, endosomes as well as mitochondria (51–53). Interestingly, the structure of donut-shaped mitochondria can be modulated by tensile force. Donut-shaped mitochondria were remodeled from tubular mitochondria under hypoxia-reoxygenation stress (54) or increased mtROS production (55). Upon examining the natural dynamics of donut-shaped mitochondria in cells without any light or pharmacological stimulation, we observed that the donut structure could be closed shortly after mitochondrial tubulation, followed by either tubule retrieval or tail-autotomy fission (*SI Appendix*, Fig. S10 *A–C*). Utilizing optogenetic mechanostimulation, we confirmed that tensile force can drive the ring closure of donut-shaped mitochondria (*SI Appendix*, Fig. S10 *D* and *E*). Our results imply that mechanical force can be an inherent regulator actively sculpting the intricate mitochondrial morphology and topology.

Cells have evolved assorted mechanisms for mitochondrial quality control to maintain mitochondrial integrity and function. Specific quality control methods are exploited depending on the nature and severity of mitochondrial dysfunction. Dysregulated mitochondrial quality control responses have been implicated in many mitochondrial diseases, such as neurodegenerative diseases, cardiomyopathies, ocular diseases, and cancer (56). Here, we found that mechanical tensile force-mediated fission promotes the biogenesis of matrix-free MDVs, implying a distinct route that can get rid of only the unwanted OMM parts while keeping desired IMM and matrix components. Several questions remain unanswered. What will happen after the recruitment of LC3B onto these matrix-free MDVs? Under what physiological or pathological circumstances will the biogenesis of matrix-free MDVs via tail-autotomy fission be employed for quality control? How can cells identify the particular need of segregating merely the mitochondrial OMM? What are the underlying mechanisms that cells steer the naturally occurring tensile force for such precise quality control? More studies are needed to address these questions.

The last decade has witnessed rapidly growing knowledge about MDVs. However, much remains unknown about the underlying mechanisms of MDV formation. Here, we identified mechanical tensile force as a player in the formation of MDVs. On the other hand, increasing interest has been drawn to the mechanoregulation of organelle activities and functions, such as those of the nucleus (57) and the Golgi (58). Along with the previous finding that compression force promotes mitochondrial fission (9), our results reveal the role of tensile force in modulating mitochondrial dynamics and MDV biogenesis, contributing to our understanding of mitochondria mechanobiology. Cells and intracellular organelles are constantly exposed to diverse extracellular or intracellular mechanical forces. It would be very interesting to study how other extracellular or intracellular mechanical cues influence cell functions via the mechanoregulation of mitochondrial fission and MDV biogenesis in various settings.

## Materials and Methods

### Cell Cultures.

COS-7 (ATCC® CRL-1651™), 3T3 (ATCC® CRL-1658™), HeLa (ATCC® CCL-2™) and NRK-52E (ATCC® CRL-1571™) cells were grown in DMEM (Thermo Fisher Scientific) supplemented with 10% FBS (fetal bovine serum, Clontech) and 1% P/S (Penicillin-Streptomycin, Thermo Fisher Scientific). U2OS (ATCC® HTB-96™) cells were cultured in McCoy’s 5A medium (Thermo Fisher Scientific) supplemented with 10% FBS (fetal bovine serum, Clontech) and 1% P/S (Penicillin-Streptomycin, Thermo Fisher Scientific). All cultures were maintained in a humidified environment at 37 °C with 5% CO_2_.

### Plasmid Construction.

The SspB variant named micro was used in our study. KIF5A-GFP-SspB, KIF1A-GFP-SspB, ePDZ-mCh-Miro1, KIF5A-GFP-LOVpep, CRY2-mCh-Miro1, and KIF5A-GFP-CIBN have been described in our previous work (12, 53). KIF5A-SspB sequence was inserted into the pEGFPN1 backbone to make KIF5A-SspB. mCh-DRP1 was obtained from Addgene (Addgene #49152), and GFP-DRP1 was then constructed by replacing mCherry with GFP using In-Fusion (Clontech). mCh-Omp25 (a.a. 111-145), DRP1K38A, TFAM-dsRed, mCh-Parkin, and GFP-LC3B were kindly provided by Prof. Xingguo Liu from Guangzhou Institutes of Biomedicine and Health, Chinese Academy of Sciences. DRP1(K38A) and TFAM sequences were inserted into mammalian expression pEGFPN1 vector to construct GFP-DRP1(K38A) by In-Fusion (Clontech) and TFAM-GFP by ligation. By In-Fusion (Clontech), iLID-tdTomato-Omp25 was obtained by inserting iLID and Omp25 into the backbone containing tdTomato. Using iLID-tdTomato-Omp25 as a backbone, iLID-tdTomato was replaced by tdTomato to make tdTomato-Omp25 using ligation. Using In-Fusion (Clontech), CRY2-tdTomato-Miro1 was made by inserting CRY2, tdTomato, and Miro1 into pEGFPN1 backbone, and iLID and tdTomato-Miro1 were fused into pEGFPN1 backbone to obtain iLID-tdTomato-Miro1. GFP-Sec61β (a.a. 1-96) was cloned by inserting Sec61β or SspB into the pEGFPN1 vector using ligation. Parkin gene was inserted into iRFP682-Smad2 (Addgene #118943) to construct iRFP682-Parkin. Restriction enzymes from InvitrogenTM were used for all the linearization of vectors and CloneAmpTM HiFi PCR Premix (Takara) was used for all the PCRs.

### Plasmid Transfection and siRNA Transfection.

Cells were plated on Poly-D-Lysine coated 35 mm confocal dishes with a hole size of 13Ø 24 h. Cells were then transfected by LipofectamineTM 3000 Reagent (Thermo Fisher Scientific) according to the manufacturer’s protocol and recovered overnight before imaging. DRP1 targeted small interfering RNAs (sense strand: 5′-UCCGUGAUGAGUAUGCUUU-dTdT-3′) as previously described (59) and MFF targeted small interfering RNAs (sense strand: 5′-CGCUGACCUGGAACAAGGA-dTdT-3′) (2, 23) were purchased from Sangon Biotech (Sangon, Shanghai, China) for the knockdown of DRP1 and MFF. Transfection of siRNA was performed using LipofectamineTM RNAiMax Reagent (Thermo Fisher Scientific) according to the manufacturer’s protocol, and cells were analyzed 48 to 72 h after siRNA transfection.

### Live Cell Imaging.

The fluorescence imaging of mitochondria, mitochondrial nucleoids, DRP1, Parkin, and LC3B was performed on an epifluorescence microscope (Leica DMi8S, Thunder Imager) equipped with an on-stage CO_2_ incubator using a 100× oil immersion objective. Images were captured with the following excitation/emission filter settings: 470 nm/510 nm for GFP, 575 nm/590 nm for mCh and tdTomato, 640 nm/700 nm for iRFP682. Blue light illumination at 480 mW/cm^2^ was used to excite both optogenetic pairs and GFP. Pulsed blue light with 200 ms exposure duration was given to the cells at 1-s intervals when sustained blue light stimulation was needed. The spatial control of blue light in the region of interest was realized by the infinity scanner on Leica DMi8S. Two-color live imaging of GFP-Sec61β and iLID-tdTomato-Omp25 was performed on a spinning disk confocal microscope (Leica DMi3000B) with 488 nm and 561 nm lasers using a 100× oil immersion objective. For this experiment, 561 nm laser (200 ms exposure, laser power 10 to 20%) was utilized to excite and image tdTomato, and 488 nm laser (200 ms exposure, laser power 10 to 20%) was used for blue light stimulation and GFP visualization. The images of ER were later processed to increase the contrast and resolution using a previously reported algorithm (60).

### Western Blot.

DRP1 antibody (Cell Signaling Technology, #8570), MFF antibody (Cell Signaling Technology, #84580) and tubulin antibody (Cell Signaling Technology) were used in this study for western blots. Total proteins of COS-7 cells seeded in 6-well plate were extracted by RIPA buffer (25 mM Tris HCl, 150 mM NaCl, 1% Triton X-100, 1% sodium deoxycholate, 0.1% SDS) containing protease and phosphatase inhibitor cocktails (Sigma) and quantified using the BCA Protein Assay (Thermo Fisher Scientific). Protein samples were separated by SDS-PAGE gels, transferred onto PVDF membranes (Bio-Rad), and blocked with 5% BSA in TBST buffer (Bio-Rad) at room temperature for 1 h. Then PVDF membranes were incubated with primary antibody at 4 °C overnight, washed in TBST buffer, and then incubated with HRP-conjugated secondary antibody (CST #7074) at room temperature for 1 h. After washing three times in TBST, the protein bands were visualized by chemiluminescence (Bio-Rad #1705060) using a ChemiDoc imaging system.

### Measurement of Mitochondrial Fission Rate.

In live cells, the division of one single mitochondrion into two daughter mitochondria was counted as one mitochondrial fission event. Taking the previously published method (2) as a reference, the fission rate of mitochondria in our study was defined as the number of fission events per square micrometer area of mitochondria. To measure the area of mitochondria, images were segmented by the trainable Weka segmentation (Fiji plugin) and then binarized. The mitochondrial area in binarized images was measured by using ImageJ Thresholding. The data were plotted and analyzed by GraphPad Prism.

### Characterization of Mitochondrial Fission Sites.

Image analysis of mitochondrial fission sites was performed with ImageJ/Fiji. For differentiating different fission sites on mitochondria, mitochondrial tubule was recognized as the thin, tubular part which was stretched out from mitochondria, and the junction was indicated as the small region on the tubule close to the spherical mitochondria body (<300 nm).

### Measurement of GFP-DRP1 Intensity.

GFP-DRP1 signal was quantified in the raw fluorescence images using ImageJ/Fiji. As described previously (2), the intensity of GFP-DRP1 was measured by drawing a ~400 nm circle at the fission site on mitochondria, and the GFP-DRP1 intensity of the same-sized circle at nonfission sites on the same mitochondrion was measured. The datasets were scaled by dividing by the mean value of GFP-DRP1 intensity of nonfission sites corresponding to fission at the junction. The graphs of GFP-DRP1 intensity were created by GraphPad Prism.

### Measurement of Average Fluorescence Intensity of Omp25, Parkin, and LC3B on the Matrix-Free MDV.

Fluorescence intensity of tdTomato-Omp25, iRFP682-Parkin, and GFP-LC3B were analyzed in the raw images using ImageJ/Fiji. At each point of time, an outline of the matrix-free MDV was drawn in the fluorescence image of tdTomato-Omp25 which marks OMM. Average intensities of tdTomato-Omp25, iRFP682-Parkin, and GFP-LC3B encircled by the outline were then measured. The graph showing average fluorescence intensity at different time points was created by GraphPad Prism.

### Line Profile Plots.

All the line profile plots were created by measuring the raw fluorescence images. Line profiles were analyzed by ImageJ/Fiji, and graphs were made by GraphPad Prism. The intensity was normalized by the formula (F-F_min_)/(F_max_-F_min_), where F refers to fluorescence intensity, and F_min_ and F_max_ are the minimum and maximum fluorescence intensity respectively along the line.

### Statistics.

All the statistical analyses were performed using GraphPad Prism. A normality test was conducted for each dataset using the D’Agostino–Pearson normality test (*P* > 0.05). For datasets considered as normally distributed, a two-sided *t* test was selected for comparing two groups. Datasets which failed the normality test were analyzed by a nonparametric test (Wilcoxon matched-pairs signed ranks test). The *P* values were indicated in the graphs: ns *P* > 0.05, *P < 0.05, **P < 0.01, ***P < 0.001, ****P < 0.0001.

## Supplementary Material

Appendix 01 (PDF)

Movie S1. **Live imaging of naturally occurring fission including tail-autotomy fission**. COS-7 cells were transfected with tdTomato-Omp25. Orange arrows indicate tail-autotomy fission events and green arrows indicate non-autotomy fission events.

Movie S2. **Live imaging of force-induced tail-autotomy fission in the right side of the cell illuminated with blue light**. COS-7 cells were transfected with iLID-tdTomato-Omp25 and KIF5A-GFP-SspB. Blue light was delivered to one side of the cell to apply mitochondria-specific mechanostimulation.

Movie S3. **Live imaging of transient force-induced mitochondrial elongation and some mitochondrial fission**. COS-7 cells were transfected with iLID-tdTomato-Omp25 and KIF5A-GFP-SspB. One 200 ms pulse of blue light was delivered.

Movie S4. **Live imaging of sustained force-induced dramatic mitochondrial elongation and frequent mitochondrial fission**. COS-7 cells were transfected with iLID-tdTomato-Omp25 and KIF5A-GFP-SspB. Intermittent blue light exposure for 5 min was delivered.

## Data Availability

Plasmids iLID-tdTomato-Omp25 (Addgene #214400) and KIF5A-GFP-SspB (Addgene #214401) are deposited at Addgene (61). All other data are included in the manuscript and supporting information.
