# Supplementary material for: Force-induced tail-autotomy mitochondrial fission and biogenesis of matrix-excluded mitochondrial-derived vesicles for quality control
Source: Proc Natl Acad Sci U S A. 2024 Mar 28;121(14):e2217019121. doi: 10.1073/pnas.2217019121 (PMC10998583; doi:10.1073/pnas.2217019121)
Supplement: Supplementary file 1 — Appendix 01 (PDF) [file pnas.2217019121.sapp.pdf]

Supporting Information for

Force-induced tail-autotomy mitochondrial fission and biogenesis of matrix-excluded mitochondrial - derived vesicles for quality control

Xiaoying Liu, Linyu Xu, Yutong Song, Zhihao Zhao, Xinyu Li, Cheuk-Yiu Wong, Rong Chen, Jianxiong Feng, Yitao Gou, Yajing Qi, Hei-Man Chow, Shuhuai Yao, Yi Wang, Song Gao, Xingguo Liu, Liting Duan*

Liting Duan
Email: ltduan@cuhk.edu.hk

This PDF file includes:

Figures S1 to S10
Legends for Movies S1 to S4

Other supporting materials for this manuscript include the following:

Movies S1 to S4

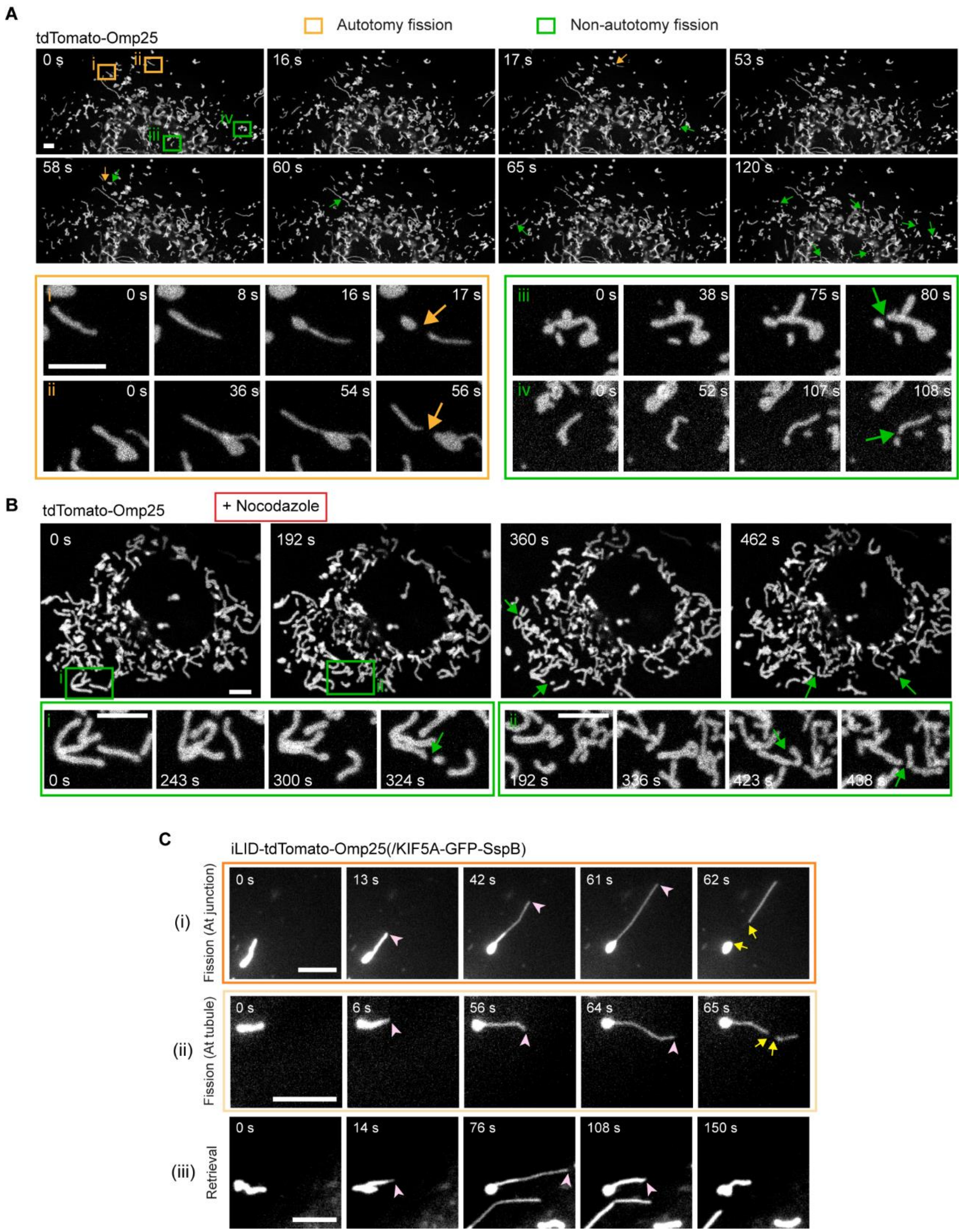

Figure S1. Spontaneous or light-gated tail-autotomy fission in COS-7 cells. (*A*) Fluorescence images of mitochondria in COS-7 cells transfected with tdTomato-Omp25. Orange arrows indicate tail-autotomy fission events, and green arrows indicate non-autotomy fission events. The zoomed-in areas show 2 autotomy fission events (orange box) and non-autotomy fission events (green box). (*B*) Disruption of microtubules inhibits naturally occurring mitochondrial tubulation and tail-autotomy fission. COS-7 cells expressing tdTomato-Omp25 were treated with 10 μM Nocodazole for 20 min

before imaging. Non-tail-autotomy fission still occurred as indicated by green arrows, while tubulation or tail-autotomy fission was not observed. (*C*) Fluorescence images of mitochondria in COS-7 cells transfected with iLID-tdTomato-Omp25 and KIF5A-GFP-SspB. Optogenetic mechanostimulation induced mitochondrial tubulation followed by tail-autotomy fission at tubule-body junction (i) or at tubule (ii), or the retrieval of tubule (iii). (Scale bars, 5 μm.)

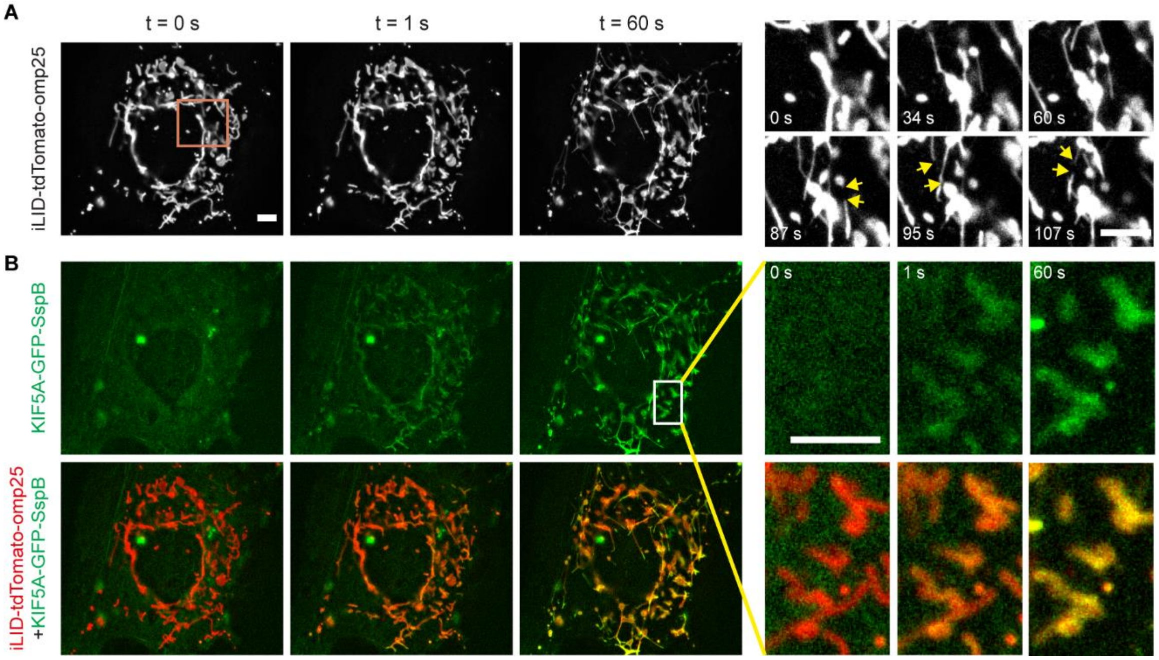

Figure S2. Light induced the recruitment of SspB-fused KIF5A onto mitochondria, hence imposing mechanical force on mitochondria. The COS-7 cell was transfected with iLID-tdTomato-Omp25 and KIF5A-GFP-SspB. Blue light was delivered to the whole cell region. *(A)* Blue light induced mitochondrial deformation and fission as shown in the area marked by the orange box, magnified on the right. Yellow arrows indicate fission events. *(B)* Blue light triggered the recruitment of KIF5A-GFP-SspB onto mitochondria, leading to the colocalization of both KIF5A-GFP-SspB and iLID-tdTomato-Omp25 signals, as shown by the zoomed-in images of the white box-indicated area. (Scale bars, 5 μm.)

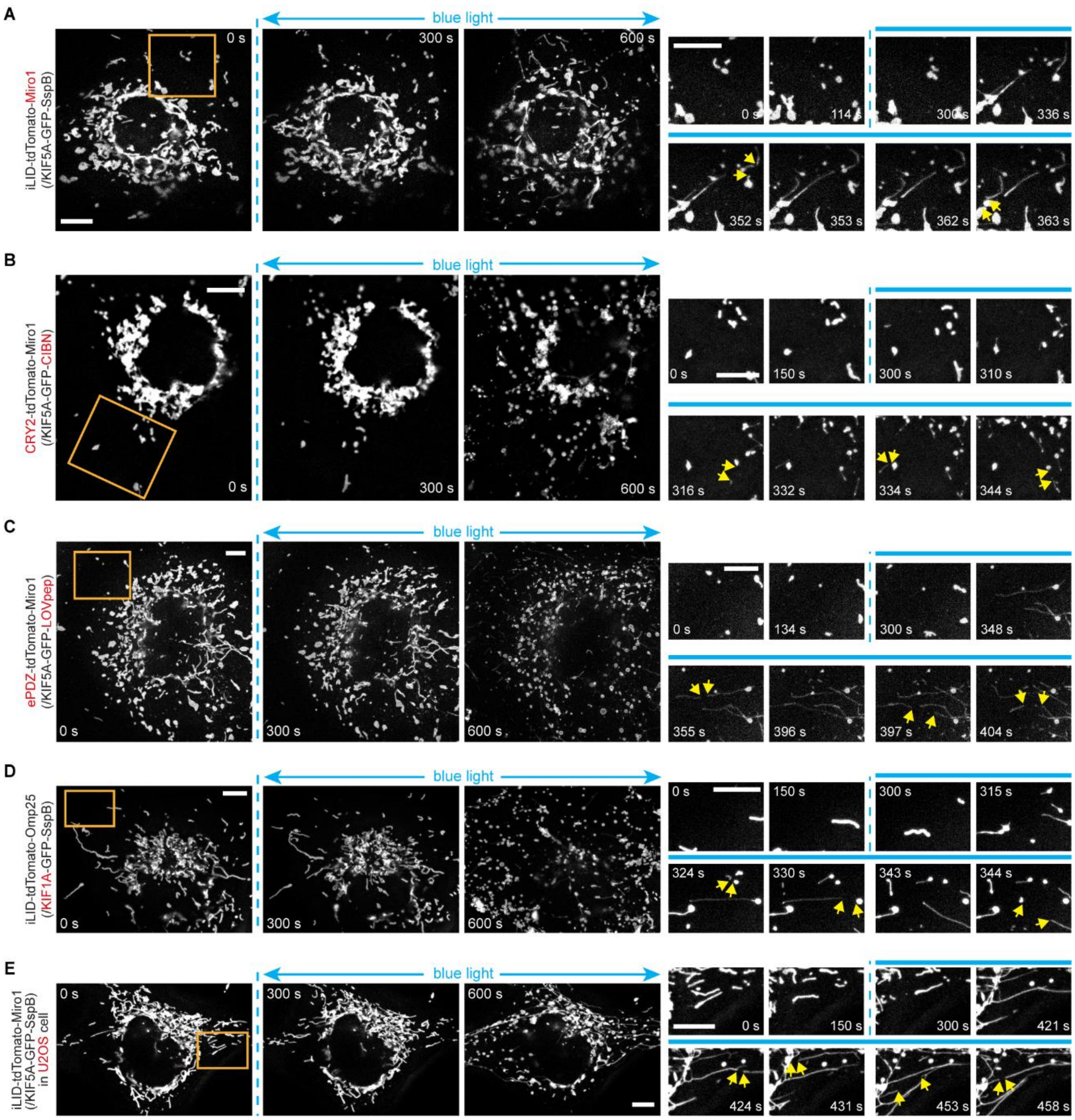

Figure S3. Force-induced tail-autotomy mitochondrial fission can be achieved by optogenetic mechanostimulator using different mitochondrial targeting sequences, optical hetero-dimers, motor proteins, and in other types of cells. In COS-7 cells subjected to 5 min intermittent blue light stimulation, mitochondria dynamics were monitored 5 min before and after the start of light stimulation. (*A*) Fluorescence images of mitochondria in COS-7 cells expressing iLID-tdTomato-Miro1 and KIF5A-GFP-SspB, where Miro1 is another mitochondrial outer membrane targeting sequence. (*B*-*C*) Fluorescence images of mitochondria in COS-7 cells expressing CRY2-tdTomato-miro1 and KIF5A-GFP-CIBN (*B*), or ePDZ-tdTomato-Miro1 and KIF5A-GFP-LOVpep (*C*), where CRY2/CIBN and ePDZ/LOVpep are another two pairs of blue light-gated optical dimerizers. (*D*) Fluorescence images of mitochondria in COS-7 cells expressing iLID-tdTomato-Omp25 and KIF1A-GFP-SspB, where KIF1A encodes another type of kinesin motor, kinesin 3. (*E*) Fluorescence images of mitochondria in U2OS cells expressing iLID-tdTomato-Miro1 and KIF5A-GFP-SspB. Optogenetic mechanostimulators using each pair of optical dimerizers, including CRY2/CIBN, iLID/SspB, and LOVpep/ePDZ, show similar efficiency of light-inducible

mitochondrial tubulation and force-induced fission. However, expression of CRY2 on the mitochondrial membrane may cause some mitochondria aggregations due to the light-gated CRY2-CRY2 oligomerization. Therefore, CRY2-integrated mechanostimulator was not utilized in the following studies. Force-induced fission is indicated by yellow arrows. (Scale bars, 10 μm.)

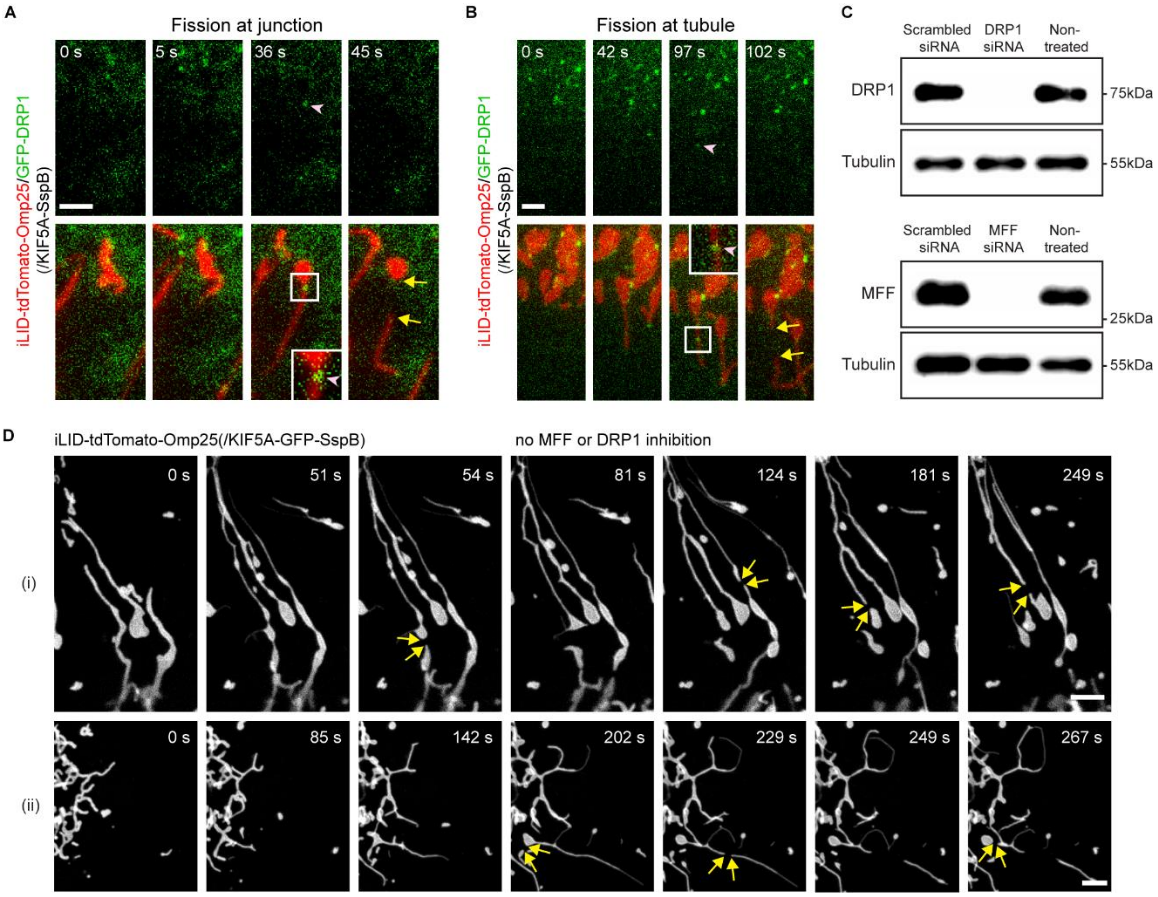

Figure S4. DRP1 and MFF participate in force-induced tail-autotomy fission. (*A*-*B*) Additional examples for DRP1 recruited on either the junction (*A*) or the tubule (*B*) during force-induced mitochondrial fission (indicated by yellow arrows). COS-7 cells were transfected with iLID-tdTomato-Omp25, GFP-DRP1 and KIF5A-SspB. (*C*) Western blot analysis shows the reduced level of DRP1 and MFF by siRNA treatment, in comparison to scrambled siRNA treatment or no treatment. (*D*) Long tubular mitochondria in COS-7 cells with functional DRP1 and MFF could still undergo force-triggered fission (indicated by yellow arrows). COS-7 cells were transfected with iLID-tdTomato-Omp25, and KIF5A-GFP-SspB without any treatment to suppress DRP1 or MFF activities. (Scale bars, 2 μm [*A*-*B*] and 5 μm [*D*].)

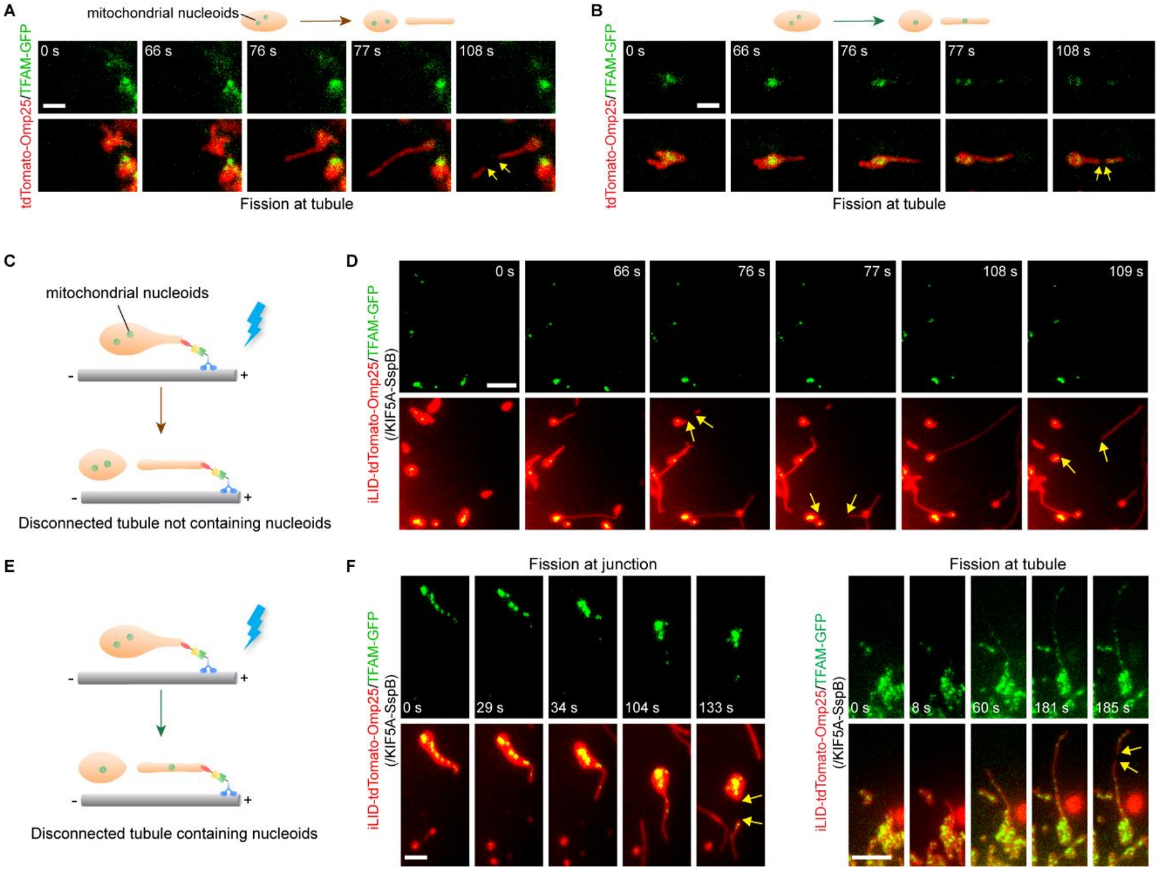

Figure S5. Examples for the generation of mitochondrial fragments with or without mtDNA by spontaneous or light-gated tail-autotomy fission. (*A*-*B*) Fluorescence images of mitochondria and mtDNA during spontaneous tail-autotomy fission (indicated by yellow arrows) at tubules which generated mitochondrial fragments without (*A*) or with (*B*) mtDNA. COS-7 cells were transfected with tdTomato-Omp25 and TFAM-GFP. (*C*-*F*) Schematic representation (*C, E*) and fluorescence images (*D, F*) of light-gated tail-autotomy fission generating mitochondrial fragments with or without mtDNA. COS-7 cells were transfected with iLID-tdTomato-Omp25, TFAM-GFP, and KIF5A-SspB. Force-induced tail-autotomy fission is indicated by yellow arrows. (Scale bars, 5 μm.)

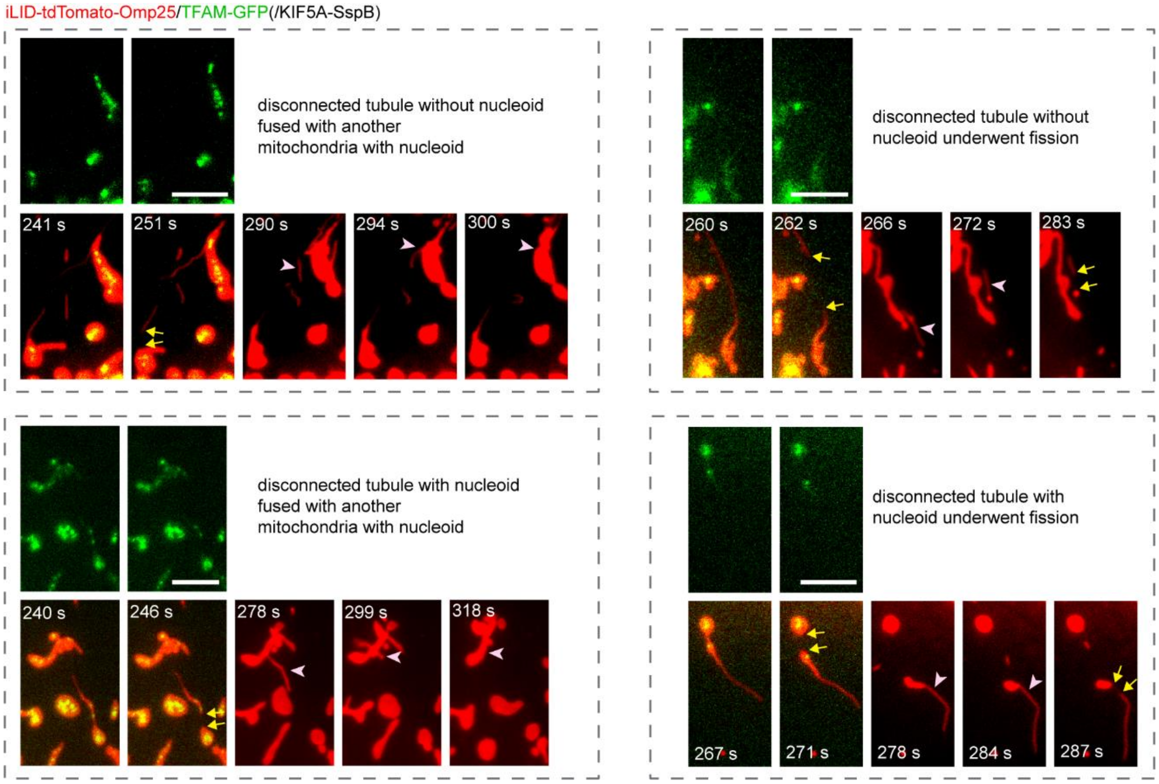

Figure S6. Different fates of mitochondrial tubule fragments generated by tail-autotomy fission. Fluorescence images of tracking mitochondria and mtDNA after light-gated tail-autotomy fission (indicated by yellow arrows). COS-7 cells were transfected with iLID-tdTomato-Omp25, TFAM-GFP and KIF5A-SspB. In the example showing disconnected tubules without nucleoids undergoing fission, the field of view is moved up between the image at time point 262s and the image at 266s to track the disconnected tubule. (Scale bars, 5 μm.)

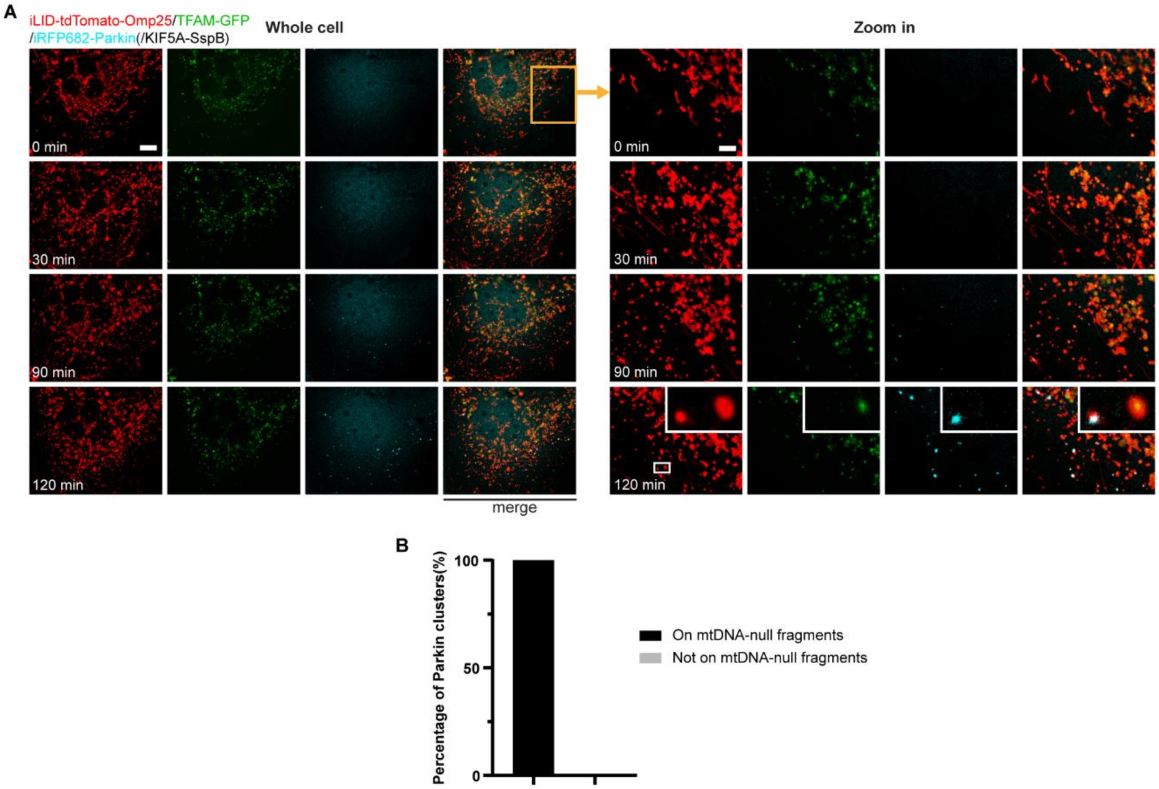

Figure S7. Force induces the formation of mtDNA-null mitochondrial fragments which recruit Parkin proteins. COS-7 cells were transfected with iLID-tdTomato-Omp25, TFAM-GFP, KIF5A-SspB and iRFP682-Parkin. (*A*) Fluorescence images of the whole cell and zoomed-in region at different time points from Fig. 6*F*. (*B*) Percentage of Parkin clusters localized on mtDNA-null mitochondrial fragments. >400 parkin clusters in 6 cells from 3 independent experiments were quantified. (Scale bars: 10 μm.)

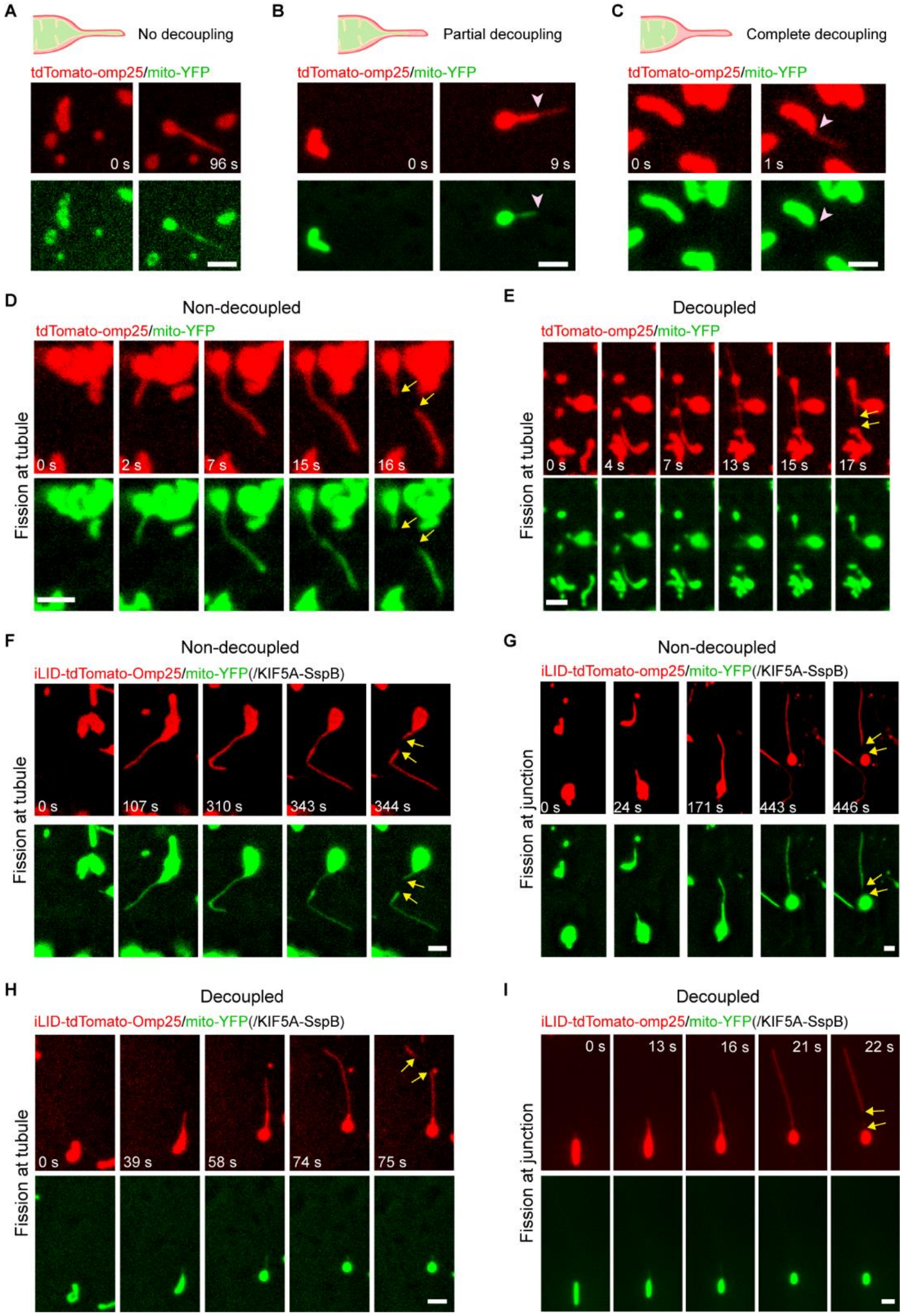

Figure S8. The decoupling of OMM and IMM and the formation of matrix-excluded mitochondrial fragments induced by spontaneous or light-gated tail-autotomy fission. (*A*-*E*) Fluorescence images showing the simultaneous deformation (*A*), partial decoupling (*B*), and complete decoupling (*C*) of OMM and IMM in the naturally formed mitochondrial tubules. COS-7 cells were transfected with tdTomato-Omp25 and mito-YFP. (*D*-*E*) Naturally occurring tail-autotomy fission at tubule with (*D*) or without (*E*) mitochondrial matrix in the disconnected tubule. (*F*-*I*) Fluorescence images showing that light-induced tail-autotomy fission generated disconnected

tubules containing (*F*-*G*) or not containing (*H*-*I*) mitochondrial matrix. COS-7 cells were transfected with iLID-tdTomato-Omp25, mito-YFP, and KIF5A-SspB. (Scale bars, 2 μm.)

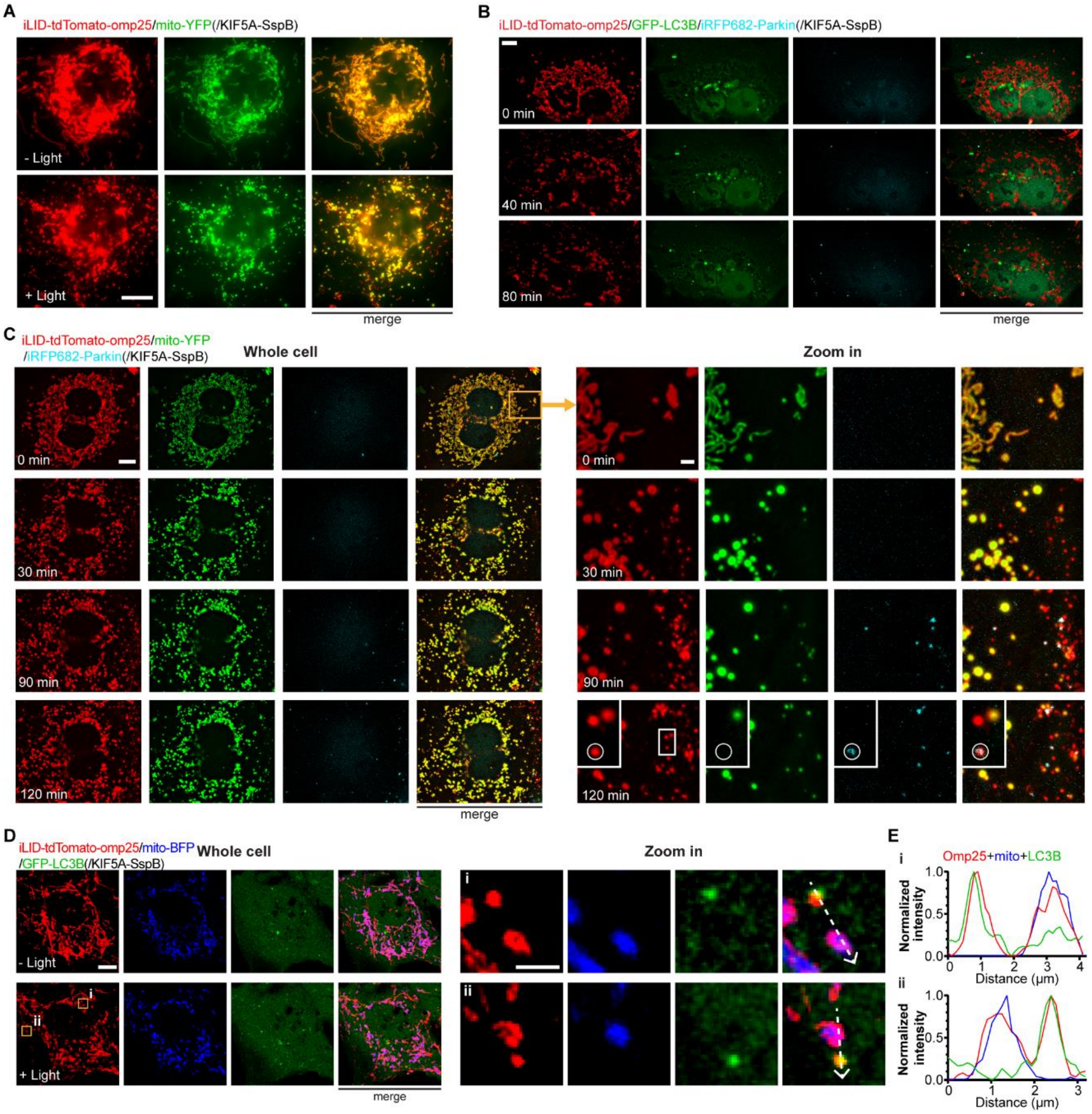

Figure S9. Matrix-excluded MDVs generated by tensile force can recruit Parkin and LC3B proteins. (*A*) Fluorescence images of the whole cell from Fig. 7*E*. COS-7 cells were transfected with iLID-tdTomato-Omp25, mito-YFP and KIF5A-SspB. (*B*) Fluorescence images of the whole cell at different time points from Fig. 7*K*. COS-7 cells were transfected with iLID-tdTomato-Omp25, GFP-LC3B, iRFP682-Parkin and KIF5A-SspB. (*C*) Fluorescence images of the whole cell and zoomed-in areas from Fig. 7*G*. COS-7 cells were transfected with iLID-tdTomato-Omp25, mito-YFP, KIF5A-SspB and iRFP682-Parkin. In *D* and *E*, COS-7 cells were transfected with iLID-tdTomato-Omp25, mito-tagBFP, KIF5A-SspB, and GFP-LC3B. (*D*) Fluorescence images showing the colocalization of matrix-free MDV with LC3B puncta. (*E*) Plot profiles showing the fluorescence intensity of iLID-tdTomato-Omp25, mito-tagBFP, and GFP-LC3B along the white dashed arrows in (*D*). (Scale bars, 10 μm for the whole cells, 2 μm for zoomed-in regions.)

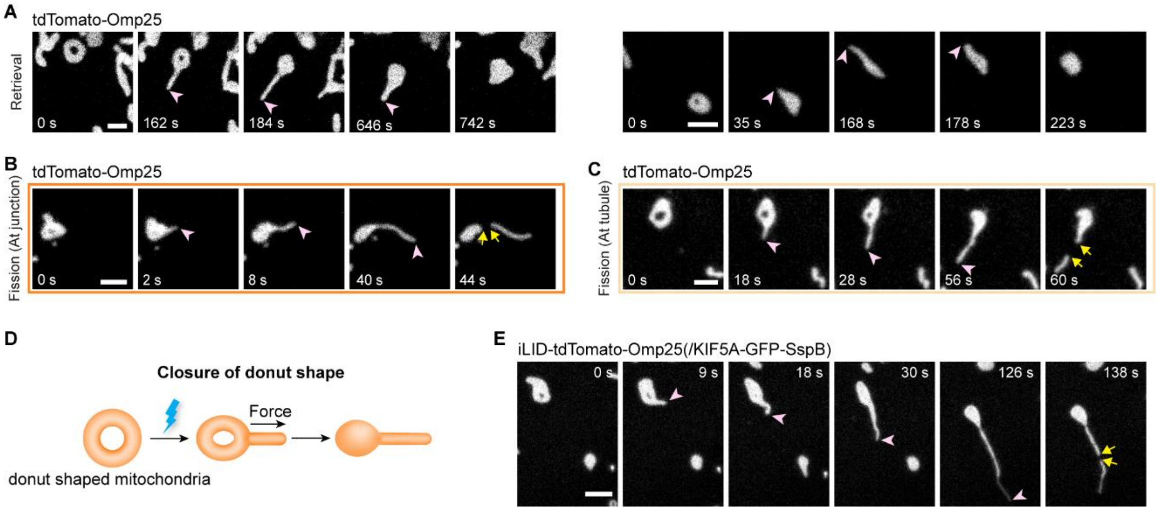

Figure S10. Tensile force can induce the ring closure in donut-shaped mitochondria followed by tail-autotomy fission. (*A*-*C*) Spontaneous mitochondrial tubulation caused the ring closure of donut-shaped mitochondria, followed by tubule retrieval (*A*), fission at junction (*B*) or fission at tubule (*C*). COS-7 cells were transfected with tdTomato-Omp25. (*D*) Illustration of the force-induced ring closure of donut-shaped mitochondrion. (*E*) Fluorescence images of donut-shaped mitochondria undergoing ring closure and then tail-autotomy fission upon light-gated mechanostimulation. Tail-autotomy fission is indicated by yellow arrows. COS-7 cells were transfected with iLID-tdTomato-Omp25 and KIF5A-GFP-SspB. (Scale bars, 2 μm.)

Movie S1 (separate file). Live imaging of naturally occurring fission including tail-autotomy fission. COS-7 cells were transfected with tdTomato-Omp25. Orange arrows indicate tail-autotomy fission events and green arrows indicate non-autotomy fission events.

Movie S2 (separate file). Live imaging of force-induced tail-autotomy fission in the right side of the cell illuminated with blue light. COS-7 cells were transfected with iLID-tdTomato-Omp25 and KIF5A-GFP-SspB. Blue light was delivered to one side of the cell to apply mitochondria-specific mechanostimulation.

Movie S3 (separate file). Live imaging of transient force-induced mitochondrial elongation and some mitochondrial fission. COS-7 cells were transfected with iLID-tdTomato-Omp25 and KIF5A-GFP-SspB. One 200 ms pulse of blue light was delivered.

Movie S4 (separate file). Live imaging of sustained force-induced dramatic mitochondrial elongation and frequent mitochondrial fission. COS-7 cells were transfected with iLID-tdTomato-Omp25 and KIF5A-GFP-SspB. Intermittent blue light exposure for 5 min was delivered.
